# Genome-wide characterization of *ALDH* Superfamily in *Brassica rapa* and enhancement of stress tolerance in heterologous hosts by *BrALDH7B2* expression

**DOI:** 10.1038/s41598-019-43332-1

**Published:** 2019-05-07

**Authors:** Ranjana Gautam, Israr Ahmed, Pawan Shukla, Rajesh Kumar Meena, P. B. Kirti

**Affiliations:** 1 Department of Plant Sciences, School of Life Sciences, University of Hyderabad, Hyderabad, 500046 Telangana India; 2 Central Sericultural Research and Training Institute, Central Silk Board, NH-1A, Gallandar, Pampore, 192121 J&K India; 3 Rajendra Prasad Central Agricultural University, Pusa-Samstipur, Bihar India; 4 Agri Biotech Foundation, Rajendranagar, Hyderabad, 500030 Telangana India

**Keywords:** Plant sciences, Plant molecular biology

## Abstract

Aldehyde dehydrogenase (ALDH) carries out oxidation of toxic aldehydes using NAD^+^/NADP^+^ as cofactors. In the present study, we performed a genome-wide identification and expression analysis of genes in the *ALDH* gene family in *Brassica rapa*. A total of 23 *ALDH* genes in the superfamily have been identified according to the classification of *ALDH* Gene Nomenclature Committee (AGNC). They were distributed unevenly across all 10 chromosomes. All the 23 *Brassica rapa ALDH* (*BrALDH*) genes exhibited varied expression patterns during treatments with abiotic stress inducers and hormonal treatments. The relative expression profiles of *ALDH* genes in *B*. *rapa* showed that they are predominantly expressed in leaves and stem suggesting their function in the vegetative tissues. *BrALDH7B2* showed a strong response to abiotic stress and hormonal treatments as compared to other *ALDH* genes; therefore, it was overexpressed in heterologous hosts, *E*. *coli* and yeast to study its possible function under abiotic stress conditions. Over-expression of *BrALDH7B2* in heterologous systems, *E*. *coli* and yeast cells conferred significant tolerance to abiotic stress treatments. Results from this work demonstrate that *BrALDH* genes are a promising and untapped genetic resource for crop improvement and could be deployed further in the development of drought and salinity tolerance in *B*. *rapa* and other economically important crops.

## Introduction

Molecular crosstalk between different stress responsive pathways seems to be an efficient survival strategy of plants to overcome and sustain against unfavorable environmental conditions during their growth and development. To adapt with the stressful conditions, plants have evolved various mechanisms by modulating the action of stress responsive genes, which might play important roles in maintaining cellular homeostasis and enhanced stress tolerance. Under the impact of environmental stresses, plants generate a series of both highly active and toxic products such as aldehydes, which hamper several cellular processes in the cells. Aldehydes are highly reactive molecules produced in different cellular and metabolic (physiological) processes such as synthesis of carbohydrates, lipids, vitamins, and amino acids^1,2^. The production of reactive aldehydes occurs through both enzymatic and non-enzymatic pathways^3^. It is a well-known phenomenon for over a decade that aldehyde molecules, when produced at disproportionate/imbalanced concentrations would have deleterious effects on plant metabolism causing lipid peroxidation, impaired cellular homeostasis, enzyme inactivation, DNA damage, and cell death^4,5^. Therefore, maintaining a concentration of reactive aldehydes at proportionate levels is crucial to cell survival and must be tightly regulated. One such group of enzymes regulating reactive aldehyde levels are aldehyde dehydrogenases (ALDHs), which act as “aldehyde scavengers” during environmental stresses. *ALDH* superfamily encompasses NAD(P)^+^ dependent enzymes that are involved in the detoxification of a broad spectrum of endogenous and exogenous aromatic and aliphatic aldehydes to non-toxic carboxylic acids^6^.

ALDHs are polymorphic enzymes having different characteristic motifs in their amino acid sequences, viz., glutamic acid active site (PROSITE PS00687), catalytic thiol (PROSITE PS00070) and the Rossmann fold^7,8^. They have been found to be widely distributed among both prokaryotes and eukaryotes, which have been classified into 24 distinct families according to *ALDH* Gene Nomenclature Committee (AGNC)^2^. In plants, 14 different *ALDH* families have been reported, out of which *ALDH11*, *ALDH12*, *ALDH19*, *ALDH2*, *ALDH22*, *ALDH23*, and *ALDH24* are exclusively found in plants^9,10^.

Previous studies suggested that the over-expression of *ALDH* genes in *Arabidopsis* plants enhanced their tolerance to different environmental stress factors^6,11–13^. An upregulation of *ALDH* in *Chlamydomonas reinhardtii* enhanced endurance during dark anoxia^14^. The increased transcript level of *At*-*ALDH3* in *Arabidopsis* was associated with improved tolerance to abiotic stresses like NaCl, and dehydration^13^. Kotochoni *et al*. (2006) showed that *Arabidopsis* plants overexpressing *ALDH311* and *ALDH7B4* genes exhibited enhanced protection against lipid peroxidation and salinity stress. In addition to their roles in different stresses, *Rf2a*, a mitochondrial *ALDH* gene has been reported for its involvement in maintaining male fertility and anther formation in maize^15^. On the basis of expression and correlation studies, four *ALDHs* genes (*ALDH3898*, *ALDH20158*, *ALDH54788*, and *ALDH11367*) were suggested to be involved in the generation of crocetin from crocetin dialdehydes from saffron in stigma^16^.

The recent accessibility of genome sequence data in the public domains provided the opportunities for convenient annotation of the genes and elucidation of the information hidden in the plant genomes. Genome-wide studies, therefore have gained importance in understanding the mechanisms related to various traits at genetic and molecular levels in plants. With a relatively small genome size (approximately 529 Mbp), *B*. *rapa* has emerged as an important model for studying the effects of polyploidy representing the ‘A’ genome of *Brassica*^17^. *B*. *rapa* commonly known as Chinese cabbage includes vegetable crops that form an essential constituent in human and animal diets worldwide, especially in Asian countries. As a result of the recent climate change scenario, *B*. *rapa* production is likely to be intensely affected by different abiotic and biotic stresses^18^. Considering its economic importance, there is a need to take steps to augment its agronomically important traits and productivity. Though the *ALDH* gene family was earlier identified in *Arabidopsis*, *Glycine max*, *Solanum lycopersicum*, *Oryza sativa*, and *Gossypium*, this gene family has not yet been comprehensively studied in commercially important *Brassica* species. The recently published genome sequence of *B*. *rapa* provided us an excellent resource for the identification and genome-wide analysis of *ALDH* superfamily in this crop species. A detailed analysis of *ALDH* genes in *B*. *rapa* has not been conducted so far with respect to the number of genes, their functional role in different stresses and in tissues of *B*. *rapa*. In an earlier study, 14 different *ALDH* genes have been reported in *A*. *thaliana*, which represented nine different *ALDH* families^19^.

To set the stage in right perspective with respect to the *ALDH* gene family in *B*. *rapa*, a comprehensive *in-silico* analysis of *BrALDH* genes that included phylogenetic relationships, promoter elements, duplication events, and chromosomal distributions was performed. We have also examined the detailed expression patterns of different *ALDH* genes under different stress conditions and tissue level in this crop species. Our gene expression study suggests the involvement of *ALDH* family genes during environmental adaptation. Also, we have identified one potential candidate *ALDH* gene based on the expression studies at transcript level and functionally characterized it by expressing it in the heterologous systems, yeast and *E*. *coli*. These observations are reported in this communication.

## Results

### Identification and sequence analysis of *ALDH* superfamily in *B*. *rapa*

A total of twenty-three candidate genes were identified to apparently encode ALDH domain containing proteins. These 23 genes were grouped into 10 different families according to the criteria established by AGNC. *ALDH3* formed the largest family in *B*. *rapa* harboring seven members (Table 1). SMART, ScanProsite and InterPro analyses revealed the characteristic presence of ALDH cysteine (PS00070, IPR016160) and glutamic (PS00687, IPR016160) residues, which are intermittently found in the proteins encoded by the *ALDH* superfamily, and were identified in 11 out of the 23 *ALDH* genes encoded proteins in *B*. *rapa*. Out of these 23, PS00070 domain is absent in proteins encoded by seven genes viz., BrALDH3H2, BrALDH3H3, BrALDH7B1, BrALDH7B2; PS000687 domain in BrALDH3I1, BrALDH6B1, and BrALDH12A1, which carried only one of the active sites. BrALDH12A2 did not contain any one of these domains, but other searches indicated that it belongs to the *ALDH* superfamily as its encoded protein possessed the alternative conserved (IPR016162-Ald-DH-N) domain.

**Table 1:** *ALDH* gene sequences retrieved from BRAD. Gene properties such as chromosome location, gene length, locus, duplication events were studied in the *B*. *rapa ALDH* genes.

Family	Gene Id	Gene Name	Chr	Gene length	Sub-genome	Signature (Locus)	Tandem duplication	Block duplication
Family 2	Br001952	*BrALDH2C1*	A03	1.506kbp	MF2	19589691–19593716	−	+
	Br018090	*BrALDH2B1*	A06	1.620kbp	LF	9850859–9853635	−	+
	Br019522	*BrALDH2B2*	A06	1.605kbp	MF2	12885987–12888783	+	+
	Br016330	*BrALDH2B3*	A08	1.614kbp	MF1	17147648–17150144	−	+
	Br024619	*BrALDH2B4*	A09	1.611kbp	LF	25223817–25226516	−	+
Family 3	Br036943	*BrALDH3H1*	Scaffold (000123)	0.858kbp	LF	179657–180927	−	+
	Br014022	*BrALDH3H2*	A08	1.449kbp	MF1	4482093–4484928	−	+
	Br032205	*BrALDH3H3*	A05	1.449kbp	MF2	13044509–13047276	−	+
	Br011511	*BrALDH3I1*	A01	1.671kbp	LF	1876841–1879683	−	−
	Br011674	*BrALDH3F1*	A01	1.446kbp	LF	1127128–1129753	−	+
	Br017753	*BrALDH3F2*	A03	1.455kbp	MF1	30292563–30294896	−	+
	Br010553	*BrALDH3F3*	A08	1.404kbp	MF2	14368085–14370471	−	+
Family 5	Br035119	*BrALDH5F1*	A07	1.593kbp	LF	25538907–25543424	−	+
Family 6	Br037214	*BrALDH6B1*	A09	1.584kbp	MF1	5026270–5030088	−	−
Family 7	Br037964	*BrALDH7B1*	A06	0.693kbp	LF	392200–393521	−	+
	Br030906	*BrALDH7B2*	A08	1.515kbp	MF1	691355–694327	−	+
Family 10	Br019528	*BrALDH10A1*	A06	1.512kbp	MF2	12530247–12533712	+	+
	Br003781	*BrALDH10A2*	A07	1.506kbp	MF2	15030838–15033835	−	+
Family 11	Br007862	*BrALDH11A1*	A09	1.491kbp	LF	31293168–31296421	−	+
	Br032110	*BrALDH11A2*	A04	1.491kbp	MF1	10851870–10854079	−	+
Family 12	Br010097	*BrALDH12A1*	A06	1.644kbp	LF	19386769–19389923	−	+
	Br029253	*BrALDH12A2*	A02	0.504kbp	MF1	26300899–26303476	−	+
Family 22	Br029596	*BrALDH22A1*	LF	1.782kbp	LF	23082205–23085442	−	−

### Phylogenetic relationship among dicot *ALDH* genes and protein properties of *B*. *rapa* ALDH proteins

A phylogenetic tree has been constructed using the 23 *B*. *rapa*, 14 *A*. *thaliana* and 19 rice (*Oryza sativa*) *ALDH* amino acid sequences to study their evolutionary relationships (Supplementary Fig. S1a**)**. The phylogenetic tree clearly depicted the relatedness of *B*. *rapa* ALDH proteins with their counterparts in *Arabidopsis* and rice. We found that eight *Arabidopsis ALDH* genes have single corresponding homologs in *B*. *rapa*, four of the *Arabidopsis ALDH* gene sequences have two homologs in *B*. *rapa* and two *Arabidopsis* amino acid sequences represent three homologs in *B*. *rapa*.

Another unrooted phylogenetic tree was constructed using the neighbor-joining (NJ) algorithm to study the evolutionary relationship among the 23 *ALDH* genes of *B*. *rapa*. As shown in Supplementary Fig. S1b, *BrALDH12A1* and *BrALDH12A2* represents the two *ALDH* genes, which diverged earlier from the rest of the *ALDH* gene sequences during the course of evolution.

In order to determine the characteristic properties of ALDH proteins, an analysis of the 23 proteins of *B*. *rapa* was performed by using Expasy’s ProtParam tool. Computed parameters of individual proteins like molecular weight, pI, GRAVY indices by ProtParam are represented in Supplementary Table S1. The molecular weights (MW) of the identified proteins ranged from 18.85 to 65.75 kDa, with 86% of these ranging from 53 to 66 kDa. We found that among all, BrALDH22A1 is the largest protein of ALDH family exhibiting a molecular weight of 65.75 kDa (with 253 amino acids). The pI analysis also revealed that almost all ALDH proteins have comparable isoelectric points in the range of 5.35 to 8.55. At isoelectric point, proteins are usually considered as stable and compact, and this allows the purification of corresponding proteins easily by isoelectric focusing method employing a proper buffer system^20^. The pI analysis showed that most of the ALDH proteins were found to be stable and compact. The GRAVY indices of 14 ALDH proteins exhibited negative values indicating their solubility. Some proteins had LCRs, which facilitate adaptations and help in protein-protein interactions and can, therefore, impact protein function^21^.

Secondary structure analysis indicated that α- helices constituted the major portion of putative BrALDH peptides followed by the β-strands (15–53%) and disordered regions (5–13%) in proteins. Apart from this, the three-dimensional structure prediction of ALDH proteins revealed different metallic (Mg^2+^ ion) and non-metallic ligands (NAD, NAI, ADP etc) interacting with their corresponding amino acid residues in 22 out of 23 ALDH proteins. BrALDH12A2 has G1P active-site pocket coordinating with the Ileu85, Phe89 residues, along with an AMP binding site, while BrALDH12A1 protein appeared to interact with NAD, NAP, NAI cofactors. BrALDH22A1 also appeared to possess two ligands, Mg^2+^ and CA for binding with Asp303, Cys68, and His226 amino acid residues. Detailed protein structures with their interacting ligands and cofactors are presented in Supplementary Fig. S2.

Three-dimensional analyses showed the presence of conserved Mg^2+^ ion in the center of the active site in the BrALDH members suggesting its crucial role in catalytic mechanisms during stress conditions. In *Saccharomyces cerevisiae*, Mg^2+^ increased the binding affinity of the cytosolic aldehyde dehydrogenase with NADP^+^ by approximately 100-fold causing a slow conformational change in the enzyme into a fully active form^22^. Using SMART analysis, we found that BrALDH2B1, BrALDH2B2, BrALDH2B4, BrALDH2B3, BrALDH5F1 exhibit low complexity regions (LCR). Except for BrALDH12A2, all the ALDH proteins possessed the aldehyde dehydrogenase domain. Some of the members possessed LuxC (acyl-CoA reductase) domain, which is known to be involved in the channelling of fatty acids to aldehyde substrate^23^. An additional AAA domain that is essential for protein degradation, regulation of gene expression and signal transduction was found in the BrALDH2B1, whereas BrALDH2B4 exhibited a BMC domain that is known to improve the carbon concentrating mechanism and an AAA domain for signal transduction and regulation of gene expression^24^. The Supplementary Table S2 summarizes the putative molecular and biological functions of BrALDH proteins along with *Arabidopsis* orthologs identified through SMART tool of the European Molecular Biology Laboratory (EMBL).

### Chromosomal locations and duplication events in *ALDH* superfamily of *B*. *rapa*

**Figure 1:**
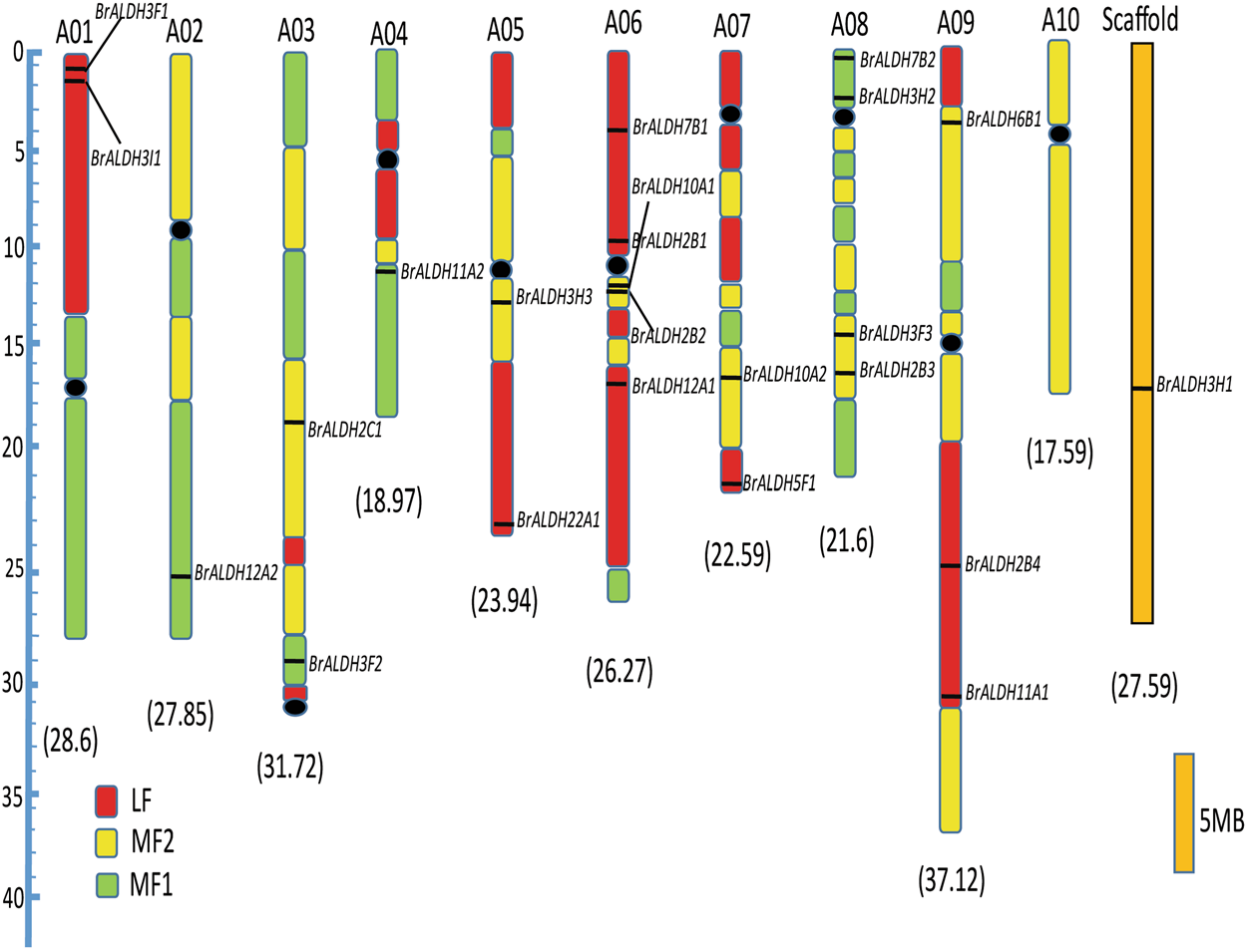
Diagrammatic representation of distribution of *ALDH* genes on the 10 chromosomes and three subgenomes of *Brassica rapa*. Chromosomal positions of each *ALDH* genes were retrieved from *Brassica* database (BRAD) using (chromosome v1.2).

All the 23 *ALDH* genes except *BrALDH3H1* were unevenly localized on all the ten chromosomes, and the location of *BrALDH3H1* gene was found to be ambiguous and has been assigned to unanchored Scaffold000123. In *B*. *rapa* chromosome complement, five *ALDH* genes were found to be located on chromosome 6, followed by chromosome 8 with four genes. The chromosome locations with their subgenome information of *B*. *rapa ALDH* genes are depicted in Fig. 1. Due to a whole genome triplication, *B*. *rapa* possesses three fractionated sub genomes i.e. LF, MF1, and MF2. Intriguingly, the LF represents the Least Fractionated subgenome containing approx 70 percent of its genes, while MF1 and MF2 represent the Medium and Most Fractionated subgenomes having retained much fewer genes^25,26^. Subgenomic studies revealed that 22 *B*. *rapa ALDH* genes were fractionated into LF, MF1 and MF2 subgenomes including 9 (41%) genes on LF, 8 (36%) on MF1, 5 (22.7%) on MF2. The duplication analysis of ALDH genes clearly indicated the presence of both tandem and block duplications. Tandem duplications were found to be present only in *BrALDH2B2* and *BrALDH10A1* genes whereas segmental (block) duplications were present in 20 *ALDH* genes. There were no tandem and block duplications observed in the *BrALDH3I1*, *BrALDH6B1*, and *BrALDH22A1* genes (Table 1). These results showed that duplication events have played an important role in the evolution of *BrALDH* gene family in *B*. *rapa*.

### Putative promoter analysis of *ALDH* genes

To gain an insight into the potential regulation mechanism of *BrALDH* genes in abiotic stress and hormonal responses of *B*. *rapa*, the *cis*-acting elements that respond to both biotic and abiotic stresses were identified in the putative promoter regions of the *ALDH*s genes. A total of fifteen putative stress elements, which included six abiotic stress and nine hormonal responsive elements were identified in the putative promoter regions of the *BrALDH* genes. Moreover, abiotic stress responsive elements like MBS (MYB binding site involved in drought inducibility), TC rich repeats (defense and stress responsiveness), HSE (heat response elements), LTR (low temperature response), and WUN (wound responsive elements) were found to be widely distributed within the putative promoter regions of these *BrALDH* genes. Further analysis revealed that hormonal responsive elements like TATC-box (gibberellin-responsive element), TCA element (salicylic acid response element), TGA element (auxin-responsive element), ERE (ethylene- responsive element), and ABRE (abscisic acid responsive element) were also found to be present in manifold copies in the promoter regions of *BrALDH* genes. Apart from this, seven genes (*BrALDH2B2*, *BrALDH2B3*, *BrALDH3F1*, *BrALDH3H1*, *BrALDH7B2*, *BrALDH10A1*, and *BrALDH6B1*) were found to contain BoxW1 motif, which responds to fungal elicitor treatment, whereas *BrALDH2B4* possessed a WUN motif that is involved in wound inducibility. In addition, we have found one to three copies of MBS elements in the promoter regions of thirteen genes (*BrALDH2B2*, *BrALDH2B3*, *BrALDH3H2 BrALDH3F1*,*BrALDH3F2*, *BrALDH3F3*, *BrALDH3I1*, *BrALDH7B2*, *BrALDH10A1*, *BrALDH11A1*, *BrALDH12A1*, *BrALDH12A2*, and *BrALDH22A1*), respectively, that responded to PEG treatments. Interestingly, among these, four genes (*BrALDH3I1*, *BrALDH7B2*, *BrALDH12A1*, and *BrALDH22A1*) were highly upregulated during abiotic stress in both shoot and root. The *BrALDH2B3* gene exhibited the maximum number of MBS elements. One common *cis*-element, TC rich repeat required for defense and stress response was also detected in the putative promoter regions of almost 17 *ALDH* genes in one or two copies, all of which displayed varied expression under salt and drought stress treatments. In the present study, we found 17 and 7 members of *B*. *rapa ALDH* superfamily genes have methyl jasmonate and abscisic acid *cis*-regulatory elements (Supplementary Fig. S3) which responded to the corresponding phytohormones. The detailed analysis of predicted stress response elements in the promoter regions of *BrALDH* genes has been presented in Supplementary Fig. S3. No significant distributions of the stress-related motifs were observed for the 23 *B*. *rapa ALDH* genes (Supplementary Fig. S4). Generally, E-values ≤ 0.05 are usually considered as significant. However in present study, E value ranged from 4.50E + 00 to 7.00E + 00, suggesting no enrichment of motifs (Supplementary Table S3)^27^.

### Differential expression pattern of *ALDH* genes in *B*. *rapa* in response to abiotic stresses and hormonal treatments

**Figure 2:**
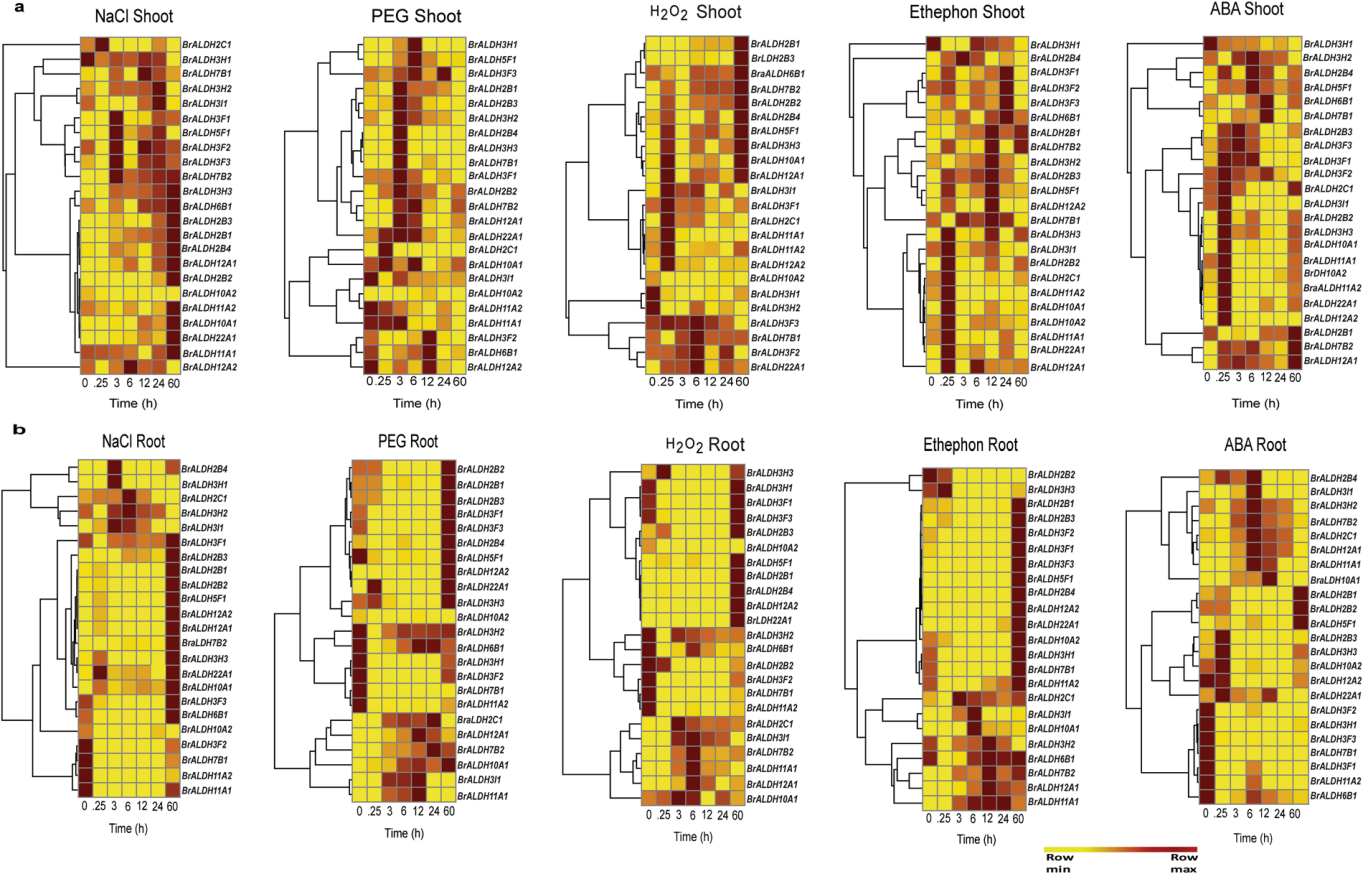
Heat map illustration of expression of *ALDH* genes in *B*. *rapa* shoot and root treated with NaCl (100 mM), PEG (10%), H_2_O_2_ (10 mM), Ethephon (1% v/v), ABA (100 µM). Mean values of the fold change values of three biological and technical replicates for the expression study were used in the qRT-PCR analysis. *B*. *rapa ubiquitin* and *actin2* were used as reference genes for normalization of gene expression.

The expression profiles of *ALDH* genes were determined in the *B*. *rapa* genome for a better understanding of their possible involvement in various stresses, mainly abiotic and hormonal treatments. For this detailed characterization, we have examined the responses of all the 23 members of the *ALDH* superfamily to three abiotic stresses, NaCl, PEG, H_2_O_2,_ and phytohormonal treatments at six different time intervals in the shoot and root tissues separately. The criterion of having fold transcript level ≥2 on the log_2_ scale was considered as upregulated. The expression pattern of *ALDH* genes in different stresses have been represented in the form of heat maps in shoot and root as shown in Fig. 2a,b. In shoots more than 61% of *ALDH* genes were observed to be upregulated under varying degrees of NaCl, H_2_O_2,_ PEG and hormonal treatments (ABA and ethephon) whereas in roots, only 30% of the genes were upregulated. Notably, some of the genes (*BrALDH2B1*, *BrALDH2B2*, *BrALDH2B4*, *BrALDH2C1*, *BrALDH3I1*, *BrALDH5F1*, *BrALDH7B2*, *BrALDH12A1*, and *BrALDH22A1*) were found to be upregulated in both shoot and root tissues in different stresses at various time intervals. This clearly suggested that this gene family might be involved in stress responses in *B*. *rapa*. The response of genes (i.e. upregulation and downregulation) towards different treatments in shoot and root have been listed in Supplementary Tables S5 and S6.

On the basis of the intensity of transcript levels at different time intervals, we have categorized the genes into immediate-early (IE), Early (E), and Late (L) responding genes for studying the detailed expression pattern of *ALDH* genes. The transcript levels of most of the *ALDH* genes were upregulated between 0.25 h to 3 h in shoots suggesting that they are IE type except under NaCl treatment. Some of the genes showed upregulation in expression patterns ranging from 2 to several 100 folds. *BrALDH5F1*, *BrALDH7B2*, *BrALDH10A1*, and *BrALDH12A1* had exhibited very high levels of expression under ABA and ethephon treatments in both shoot and root, respectively. The *ALDH* genes that exhibited expression levels between 2–10 fold were designated as low expressing and between 10–30 folds as moderately expressive, and genes with more than 30 folds increase being designated as highly expressive. The genes, *BrALDH2B2*, *BrALDH3I1*, and *BrALDH7B2* exhibited the expression of up to 100 folds under the highly expressive category. *BrALDH3H3* and *BrALDH5F1* evidenced the transcript levels up to 30 folds and were designated as moderately expressive while the others (*BrALDH2B3*, *BrALDH2B4*, *BrALDH3F1*, *BrALDH3F3* and *BrALDH12A1*) were considered as low expressive having expression levels less than 10 fold.

In addition, the expression of *BrALDH7B2* was found to be upregulated in all stress treatments from 0.25 h to 6 h followed by downregulation at 12 h and 24 h and then showed a rebound in its elevated expression level again at 60 h time point. Moreover, *BrALDH7B2* exhibited a similar expression pattern in ABA and ethephon treatments, whereas the transcript level of *BrALDH7B1* was consistent showing no enhancement in expression level in all stresses being in the same family. This could be due to high redundancy in the function of these two genes. However, the expression of this gene during NaCl treatment was found to be in contrast with the expression level under ABA stress condition in shoots. The level of expression of *BrALDH5F1*, *BrALDH10A1*, and *BrALDH12A1* was upregulated at 0.25 h followed by downregulation at subsequent time intervals in shoots under ethephon treatments. On the other hand, several *ALDH* genes were found to be late responding showing expression at 60 h in shoots during NaCl stress. However, the level of expression of the *ALDH* genes was found to be high in NaCl as compared to all other treatments. *BrALDH11A1* and *BrALDH11A2* showed significant upregulation at 0.25 h and then lower or no transcripts until the end of the time period. There was an overlap in the expression of the 23 *ALDH* genes (i.e. upregulation and downregulation) in both root and shoot tissues at various time intervals. This overlap of upregulated **(**Supplementary Fig. S5) and downregulated genes (Supplementary Fig. S6) is shown in the form of Venn diagrams. To investigate whether the stress treatment was accurately given, we compared the expression of some stress marker genes in shoot of treated and untreated samples at 6 h. We found the stress marker genes showed relative abundance of transcripts in the treated samples compared to untreated samples (Supplementary Fig. S7).

### Tissue-specific expression profiling of *ALDH* genes

**Figure 3:**
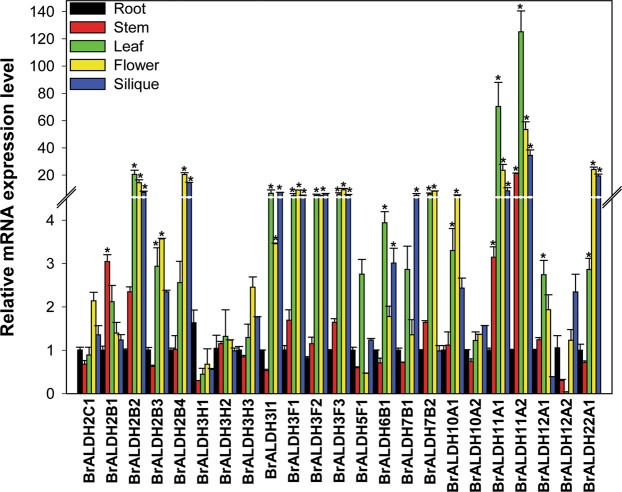
qRT-PCR analysis of *BrALDH* genes during native state in different tissues (root, stem, leaves, flower and siliques). Root expression of each *ALDH* genes was used to calculate relative expression of the *ALDH* in stem, leaves, flower and silique. Three biological and technical replicates were used in the present study. Statistical significance was done using one way ANOVA with Duncan’s Multiple Range Test (DMRT) at P ≤ 0.05 (represented by *).

To further investigate the native expression patterns of *ALDH* genes in *B*. *rapa*, we have performed the expression analysis of all the 23 *ALDH* genes in different tissues of *B*. *rapa* plants (shoot, root, leaf, flower and siliques). The relative expression of individual members of *BrALDH* genes was computed with respect to their expression in the roots (Fig. 3). Of the 23 genes identified, the expression of 10 *ALDH* genes was significant in leaves and stem as compared to roots, whereas other genes did not show any expression under the non-stress conditions. Particularly, *BrALDH2B1*, *BrALDH2B2*, and *BrALDH11A2* showed expression only in stem. The relative expression of *BrALDH3F1*, *BrALDH3F2*, *BrALDH3F3*, and *BrALDH11A2* was found to be high in leaves. The expression of *BrALDH11A2* was found to be significantly high in both leaf and stem. The expression of *ALDH* genes was also higher in flowers and siliques as compared to roots. Nevertheless, there were no differences in the relative transcript levels of *BrALDH* family members 5, 7 and 10 as observed in different tissues as compared to roots.

### Overexpression of *BrALDH7B2* improves tolerance to salt and oxidative stress in *E*. *coli*

We analyzed the functional role of highly expressed *BrALDH7B2* gene under different stress conditions in heterologous systems like *E*. *coli* and yeast. For this study in *E*. *coli*, *BrALDH7B2* was cloned into the expression vector pET28a and transformed into the bacterial cell line BL21 (DE3). The over-expressed protein resulted in the production of a band of ~55 kDa in SDS-PAGE analysis. SDS-PAGE detection showed the overproduction of recombinant BrALDH7B2 protein in cell extracts from pET28a: *BrALDH7B2*, but not in cells carrying the control (pET28a) plasmid (Supplementary Fig. S9a).

**Figure 4:**
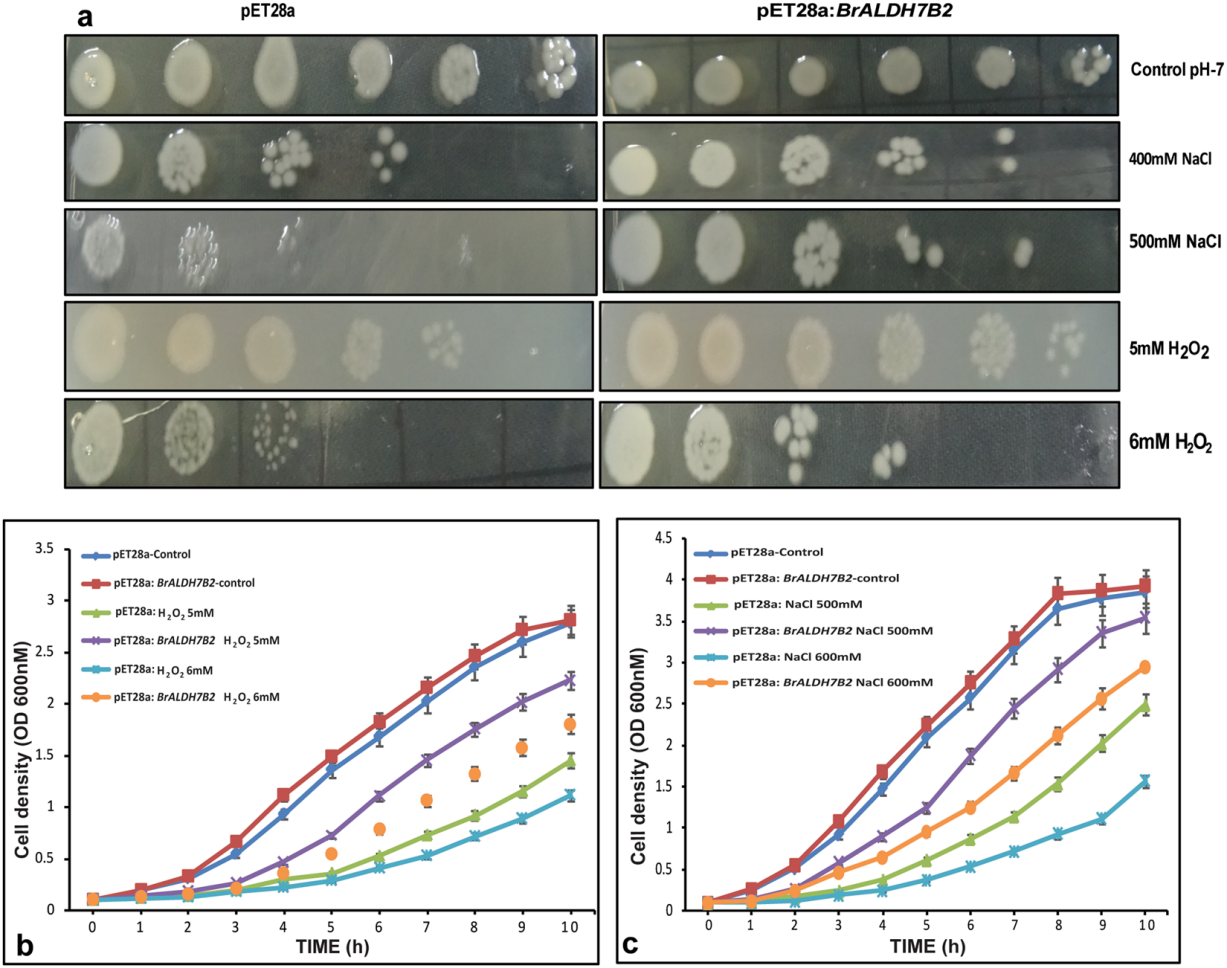
Overexpression of *BrALDH7B2* in pET28a in *E. coli*: (**a**) Spot assay showed better tolerance of recombinant BL21/*BrALDH7B2*. (**b**,**c**) Growth kinetics study of BL21/pET28a and BL21/*BrALDH7B2* during abiotic stress. Bacterial cells were grown in LB medium having 400/500 mM NaCl or 5/6 mM H_2_O_2_ treatments. Cells were monitored for 10 h at 1 h interval at 37 °C. Mean values and standard deviations of growth of three independent experiments were plotted in the form of graph.

The effects of NaCl and H_2_O_2_ treatments on the *E*. *coli* cell viability was evaluated by growth curves. The *E*. *coli* cells harboring pET28a: *BrALDH7B2* construct were found to exhibit significant tolerance to NaCl and H_2_O_2_ stresses compared to the cells carrying only the empty pET28a vector (Fig. 4b,c). The spot assay also showed similar results when the recombinant plasmids were expressed in *E*. *coli* (Fig. 4a) and this demonstrated that BrALDH7B2 could enhance the viability and survival of *E*. *coli* cells under salt and oxidative stress treatments in heterologous systems. Hence, it seems that this gene encoded protein might act in the prokaryotic cells by degrading toxic aldehydes generated in the cell during NaCl or H_2_O_2_ treatments and thus aids in improving their tolerance to corresponding stresses.

### *BrALDH7B2* enhanced abiotic stress tolerance of the eukaryotic system, *Saccharomyces cerevisiae mutant* W_3_O_3_-1-A

**Figure 5:**
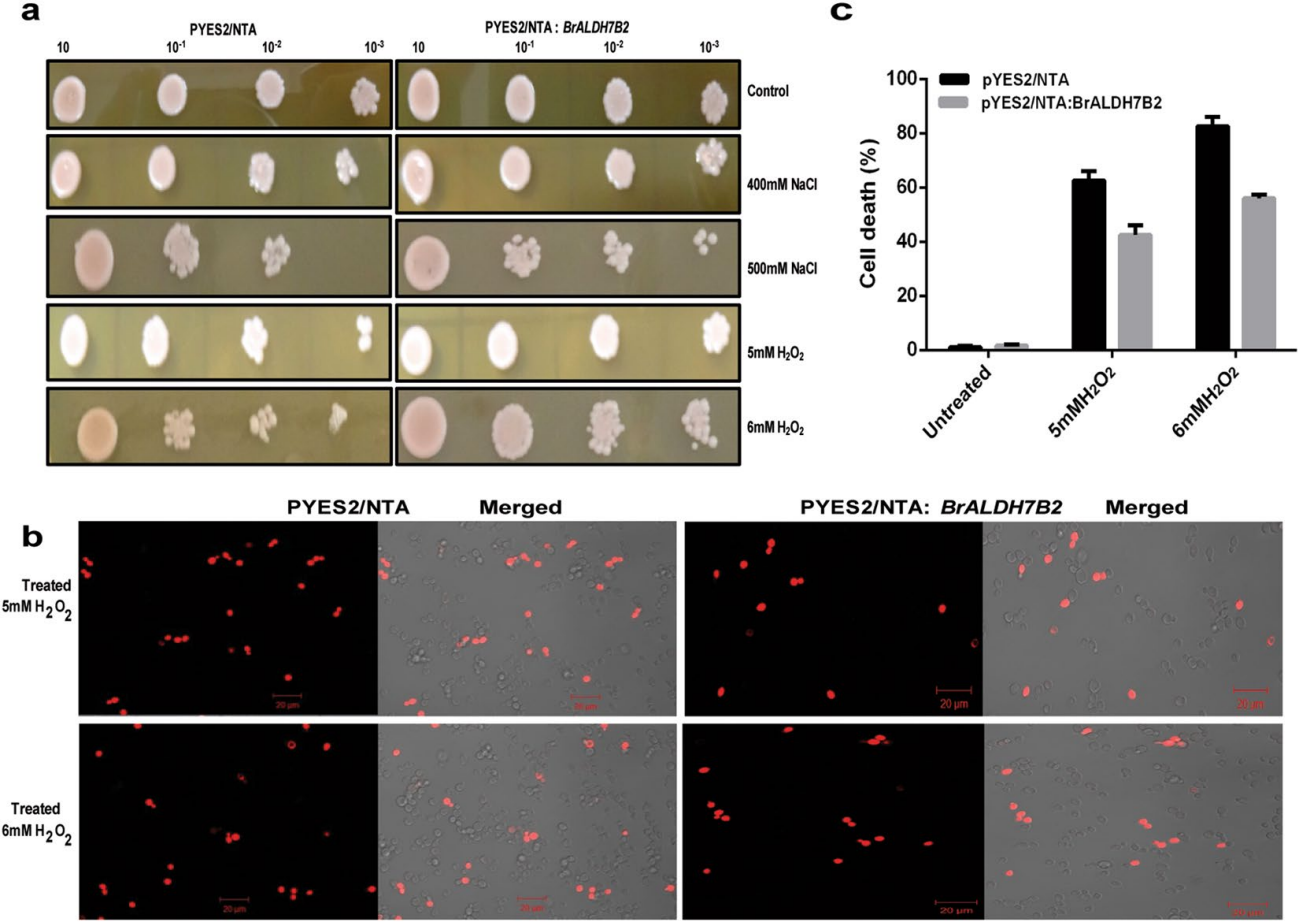
Heterologous expression of *BrALDH7B2* in yeast mutant W_3_O_3_-1-A. (**a**) Comparison of cell survival assays of *S* . *cerevisiae* mutant W_3_O_3_-1-A expressing PYES2/NTA and PYES2/NTA: *BrALDH7B2* under different abiotic stress treatments up to 10^−3^ fold dilutions. W_3_O_3_-1-A cells at OD600 = 0.5, were spotted on YPDA medium (Yeast potato dextrose agar) supplemented with 400/500 mM NaCl and 5/6 mM H_2_O_2_ and 2% galatose. (**b**) Pictorial depiction of PI treatment. Images were observed under Laser scanning confocal fluorescence microscopy of yeast cells carrying PYES2/NTA and PYES2/NTA: *BrALDH7B2* in 5 mM and 6 mM H_2_O_2_ at λem = 620 nm and λexc = 530 nm. Dead cells were stained red colour.Scale bar = 20 μm; (**c**) Quantification of dead cells under confocal microscopy in each field. Three different fields of view from each slide were chosen randomly. More than 300 cells from three independent experiments were counted and plotted in the form of graph.

The *BrALDH7B2* has also conferred tolerance to abiotic stress treatments in *Saccharomyces cerevisiae* mutant W_3_O_3_-1-A (that possesses a ybp1-1 mutation, which increases sensitivity to oxidative stress). Heterologous expression of *BrALDH7B2* in yeast mutant cell line W_3_O_3_-1-A was confirmed by Immunoblotting. The W_3_O_3_-1-A yeast mutant carrying the vector pYES2/NTA: *BrALDH7B2* after the induction of GAL promoter, expressed a fusion protein BrALDH7B2 with His-tag of ~55 kDa after 2 h of induction, which was absent in the cells transfected with pYES2/NTA alone. Maximum expression of the recombinant polypeptide was obtained after 3 h of induction as shown in Supplementary Fig. S9b, when exposed to anti-His antibodies during immunoblotting. To further validate the role of *BrALDH7B2* in imparting tolerance to salinity and oxidative stresses, the yeast system was used. In the control media, yeast transfectants showed a similar growth pattern compared to the transfected cells carrying the empty vector. However, treatments with 400 mM NaCl and 5 mM H_2_O_2_ exhibited significant differences between the W_3_O_3_-1-A mutant yeast cells having an empty vector, pYES2/NTA: *BrALDH10A2* and pYES2/NTA: *BrALDH7B2* constructs. When the concentration of the stress agents was increased to 500 mM NaCl and 6 mM H_2_O_2,_ the yeast transfectant cells harboring pYES2/NTA: *BrALDH7B2* were found to exhibit reduced sensitivity and enhanced survival (Fig. 5b) as compared to both controls i.e., cells with empty vector pYES2/NTA (Fig. 5a) and pYES2/NTA: *BrALDH10A2* (Supplementary Fig. S9c) showing that the expression of *BrALDH7B2* enhanced stress tolerance in yeast during NaCl and H_2_O_2_ stress treatments.

Propidium iodide staining under normal conditions resulted in only 1% of the nuclei of yeast cells stained by PI, which can enter only cells with the damaged cell membrane. However, the number of non-viable cells stained with PI increased in the presence of 5 mM and 6 mM H_2_O_2_ stress conditions. In the presence of 6 mM H_2_O_2_, yeast cells with empty vector showed significant increase in mortality as compared to pYES2/NTA: *BrALDH7B2* yeast cells (Fig. 5b,c) showing that the expression of the *ALDH* gene enhanced yeast mutant cell viability significantly.

## Discussion

A comprehensive investigation of plant responses towards abiotic and biotic stresses not only provides information, which can be used for advancement in agriculture but also offers the freedom for sustainable agriculture. In the present investigation, we have made an attempt to systematically identify and characterize the *ALDH* superfamily in *B*. *rapa* including the expression patterns of *ALDHs* genes in a tissue-specific manner and across several abiotic and hormonal treatments. Recent availability of the complete sequence of *B*. *rapa* genome has helped in the annotation and identification of 23 *ALDH* genes located on 10 chromosomes in this crop species. The *ALDH* gene superfamily appeared to expand in *B*. *rapa* genome as compared to *Arabidopsis* belonging to families 2, 3, 5, 6, 7, 10, 11, 12, and 22. We found these genes to be unevenly distributed on all the 10 chromosomes with a maximum number of genes located on chromosome numbers 6 and 8, except *BrALDH3H1*, which is located on the Scaffold000123. This could be due to some constraints in the sequencing technology^28–30^. In the previous studies, it has been shown that tandem duplication, segmental duplication, and whole genome duplication mechanisms resulted in the expansion of gene families and the genomes in the plants^25^. Among the three events, whole genome duplication through meso-polyploidization^31^ has been well established during the time of evolution in Brassicaceae^32^. However, in the present study, the *ALDH* superfamily in *B*. *rapa* has probably undergone two tandem duplications associated with twenty segmental duplications. Our results are in consistent with a recent report on tandem duplication for expansion in these gene families in rice and grapes^33^. In addition to the above, *Arabidopsis* genome carried only 14 *ALDH* members; accordingly, the whole genome triplication (WGT) might have resulted in 42 genes in *B*. *rapa*. However, only 23 *ALDH* genes were detected in this study suggesting the possibility that more than 45% of the redundant genes were lost during chromosome fractionation and reshuffling after WGT during re-diploidization^32^.

In the present study, we have performed a detailed expression profiling of *ALDH* genes in native and under different stress treatments. Among the *BrALDH* superfamily, members of the family 2 (*BrALDH2B1*, *BrALDH2B2*, *BrALDH2B4*, *BrALDH2C1*), family 7 (*BrALDH7B2*), family 3 (*BrALDH3I1*), family 5 (*BrALDH5F1*), family 12 (*BrALDH12A1*), and family 22 (*BrALDH22A1*) were significantly upregulated during different stress treatments (NaCl, PEG and H_2_O_2_) as compared to their control counterparts suggesting their involvement in molecular pathways related to stress responses. Quantitative gene expression analysis suggested that approximately 75% and less than 30% of *ALDH* genes were differentially expressed under hormonal and abiotic treatments in shoots and roots, respectively. This clearly suggests that *ALDH* genes are more functional in shoots as compared to roots and may be involved in protecting the photosynthetic apparatus of plants^34,35^. Subsequent upregulation of *ALDH* genes in *B*. *rapa* during PEG treatment suggests that these genes might be functioning in limiting water conditions to maintain cell homeostasis Recently, He *et al*.^33^ had also investigated the role of *ALDH* members in *Gossypium raimondii* and found that seven genes were upregulated in response to water deficit. During NaCl treatment, maximum of the *BrALDH* genes were significantly upregulated at 60 h showing up to ≥100 fold transcript level in shoots suggesting their involvement in improving tolerance to salinity stress. Transgenic *Arabidopsis* overexpressing *VpALDH2B4* (from *Vitis pseudoreticulata*) showed enhanced resistance to salt stress and mildew pathogen^36^. The observations in the present study strongly suggest that *BrALDH* genes play prominent roles in salinity, oxidative and drought stresses and therefore, could contribute to plants adaptation to abiotic stress conditions.

The significant upregulation of transcripts of *BrALDH2C1* during later stages of stress suggests that this gene functions in response to high ROS level and may be involved in regulating ROS levels during severe stresses at later stages. The phytohormones are well known for their vital role as signaling molecules during adaptive responses to different stresses. We found *B*. *rapa* genes particularly, *BrALDH5F1*, *BrLDH7B2*, *BrALDH10A1* and *BrALDH12A1* genes were highly upregulated during ABA treatment, which is consistent with the *AtALDH3* expression in *Arabidopsis* during ABA stress^37^. Enhancement of *BrALDHs* expression during treatment of phytohormone ABA indicates that these genes are induced to counteract the harmful aldehydes generated by excessive ROS formation in plants cells in stress conditions. It appears that *ALDH* genes are the targets of the ABA signaling pathways involved in stress response. The previous reports on *Arabidopsis ALDH3I1* and *ALDH7B4* also suggested their involvement in ROS detoxification and lipid peroxidation inhibition^12,13^. A similar pattern of upregulation of *BrALDH5F1*, *BrALDH10A1* and *BrALDH12A1* genes was also noticed during ethephon treatment in the present investigation. The altered responses of *ALDHs* to ethephon treatment pinpoint to their involvement during biotic stress and perhaps they might also be acting as targets in salicylic acid (SA) and jasmonic acid (JA) pathways. Recently, inactivation of two genes *MoKDCDH* and *MoP5CDH* (member of family four aldehyde dehydrogenase genes superfamily) in the rice pathogen *Magnaporthe oryzae* by RNAi- method caused reduction in conidia formation and vegetative growth of the mutant fungal strains^38^. It would be interesting to see the expression of *ALDH* genes during SA and JA treatments and this study might help in a better understanding of interaction network of these phytohormones. Kirch *et al*.^8^ have suggested that the *ALDH* genes are involved in several pathways and also their regulation comprises of diverged signal transduction pathways. Few genes like *BrALDH3H1*, *BrALDH3I1*, *BrALDH3F2*, and *BrALDH6B1* have displayed low or no expression under ABA and ethephon treatments suggesting their lack of involvement during stress.

Our analyses of *ALDH* during native conditions indicated their widespread expression in leaves, flower and silique. Furthermore, our ALDH expression analysis assumes that ALDH proteins are involved in the oxidation of aldehydes to meet the energy demand for various metabolic or biosynthetic requirements in leaves. In plants, the expression of *ALDH* members was found to be variably widespread and developmentally regulated^39,40^. We have observed wide variation in the occurrence of stress elements present in the putative promoter regions of *BrALDH* genes and the existence of various *cis*-regulatory elements and the present analysis corroborated the expression profiling of the *ALDH* superfamily in *B*. *rapa* implying their significant roles in different biotic and abiotic stress mechanisms in a way similar to other systems like *Arabidopsis* and rice. Interestingly, among these differentially expressed genes, *BrALDH7B2*, a member of *antiquitin* responsive gene family has exhibited higher transcript levels as compared to other *ALDH* genes in all stress conditions. The promoter region of *BrALDH7B2* was also enriched in TGACG, HSE and LTR elements, which is consistent with the promoter analysis results of *ALDH7B4* of *Arabidopsis*^41^. Recently, a functional analysis of the *7B4* promoter of *ALDH* gene of *Arabidopsis* showed that its expression in seeds involved both ABA and lipid signalling pathways^41^.

In an attempt to validate our expression data, we further investigated the functional role of the promising *BrALDH7B2* gene *in-vivo*. Towards achieving this, we have expressed this *ALDH* gene in *E*. *coli* and in a mutant W_3_O_3_-1-A of the unicellular eukaryotic model system, yeast having a ybp1-1 mutation (Yap1p-binding protein responsible for oxidative stress tolerance)^42^. We found that the overexpression of *BrALDH7B2* protein in *E*. *coli* cells potentiated multiple stress tolerance in both bacterial spotting and cell survival tests. Similarly, increased tolerance was also achieved in the yeast mutant during cell survival assays and in Propidium iodide staining during oxidative stress conditions. This suggests that *BrALDH7B2* could also be involved in detoxification through reactive aldehyde scavenging even in eukaryotic cells. In line with our results, the ectopic expression of soybean *GmALDHTP55*, a homolog of the *antiquitin* gene in *Arabidopsis* and *Nicotiana* increased tolerance to salinity, water deficit, and oxidative stress during germination and plant growth^6^. These findings show that both prokaryotes and eukaryotes might have conserved and close resemblance during cellular responses during stress conditions^43^.

In conclusion, our present investigation provided detailed information on the genome-wide analysis and the expression profiles of *ALDH* genes during stress responses in *B*. *rapa*. Segmental duplication and whole genome duplication have contributed to the expansion of the *ALDH* gene family in this crop species. Our expression analysis has also highlighted the potential functions of *BrALDH* genes in abiotic stresses. Overexpression of *BrALDH7B2* in *E*. *coli* and yeast showed enhanced tolerance to H_2_O_2_ and NaCl.

Based on the expression patterns, some candidate genes have been identified, which could be implicated in salinity, drought and oxidative stress tolerance in crops. Interestingly, we have observed the differential expression of some *ALDH* genes like *BrALDH2B4*, *BrALDH3I1*, and *BrALDH7B2* in different stresses and future detailed characterization of these genes would shed light on physiological adaptation and understanding of aspects of the regulatory mechanisms in stress tolerance in agronomically important *Brassica* and other crops.

## Material and Methods

### Database searches for sequence retrieval and identification of *ALDH* genes

The *ALDH* gene family members of *B*. *rapa* were identified using the SWISS-PROT tool analysis of the *B*. *rapa* database using the keyword “ALDH” (http://brassicadb.org/brad/index.php). Protein and CDS (Coding DNA Sequences) sequences of *ALDH* members of *B*. *rapa* were retrieved using the *Brassica* database BRAD (http://brassicadb.org/brad/geneFamily.php, V1.5) and homology searches using NCBI Blast (https://blast.ncbi.nlm.nih.gov/Blast). The resulting protein sequences were further confirmed for the existence of the conserved ALDH domain - PF00171 using SMART tool of Embel (http://smart.embl-heidelberg.de/), NCBI CDD and Pfam (http://pfam.xfam.org) and IPR015590 using InterPro, the integrative protein signature database (https://www.ebi.ac.uk/interpro/). To confirm the presence of *ALDH* cysteine active site (PS00070) and glutamic acid active site (PS00687), web tool PROSITE scan (http://prosite.expasy.org) along with InterPro web tool (https://www.ebi.ac.uk/interpro/) was used. The *Arabidopsis* and *O*. *sativa* ALDH protein sequences were obtained using TAIR (https://www.arabidopsis.org/) and RGAP-DB v7 (http://rice.plantbiology.msu.edu/) for analysis.

### Phylogenetic analysis and Structural features of ALDH Proteins *of B*. *rapa*

Multiple sequence alignment of ALDH protein sequences identified from the Brassica database (BRAD) of *B*. *rapa*, *Arabidopsis* and *O*. *sativa* from TAIR was carried out using clustalW. For studying the evolutionary relationship, we have constructed a phylogenetic tree based on the neighbor-joining (NJ) algorithm with MEGA v.7 using 1000 bootstrap replicates.

For predicting the physical properties of proteins such as molecular weight, protein length and isoelectric point freely available online tool, Expasy ProtParam (https://web.expasy.org/protparam/) was used. The GRAVY (Grand Average of Hydropathy) of the ALDH proteins was also determined using Expasy ProtParam. GRAVY values indicate hydropathy index, which ranges from −2 to +2 for most proteins, while the negative values indicate the hydrophilic nature of proteins. And the positively rated ones are characterized as more hydrophobic. For a better understanding of the ALDH proteins in *B*. *rapa*, we have performed a secondary structure composition analysis. Phyre2, which was a Protein Homology/AnalogY recognition Engine v.2, was utilized to predict the three -dimensional secondary structures of the peptides^44^ and 3D ligand Site for the analysis of metal/non-metal ligands and their ligand binding residue^45^.

### Chromosomal distribution and gene duplication events of *BrALDH* genes

Subgenome information including the chromosomal positions of individual *ALDH* genes was obtained from *Brassica* database using (chromosome v1.2). The physical positions of the 23 *B*. *rapa ALDH* genes on 10 chromosomes were located manually. Gene duplication information was retrieved from the PLAZA3.0 dicot database.

### Prediction of putative *cis*-elements and their enrichment analysis

To identify the putative stress responsive *cis*-elements, sequences ≤1 kb upstream to the start site of each of the individual *ALDH* genes were retrieved from BRAD genome browse tool. The sequences were then submitted to the Plant Care database (http://bioinformatics.psb.ugent.be/webtools/plantcare/html/) to predict the putative promoter elements of *BrALDH* genes. Motif enrichment analysis of the identified *cis*-elements in all 23 *ALDH* genes was performed using CentriMo Enrichment Local Motif tool of MEME software (Version 5.0.4). During this analysis, a set of two hundred four genes of the *B*. *rapa* genome that are not involved in stress was used as a control for sequence comparison of the motifs^27^.

### Plant material and growth conditions

*B*. *rapa* cv. YID-1 (Pusa Gold) seeds were obtained from Dr. S.R. Bhat of the National Research Center on Plant Biotechnology, IARI, New Delhi, India. Seeds were surface sterilized with 70% ethanol for 1 min followed by a treatment with 0.1% aqueous mercuric chloride for 5 min and three times washing with sterile double distilled water. Seeds were germinated on solid half strength MS medium under a 16 h light/8 h dark photoperiod.

### Stress Treatments

To investigate the responsiveness of *BrALDH* genes to different abiotic and hormonal treatments, one-week-old seedlings of *B*. *rapa* were subjected to NaCl (100 mM), PEG (10%), H_2_O_2_ (10 mM), Ethephon (1% v/v), ABA (100 µM) treatments. The shoot and root samples were collected separately at different time intervals, viz. −0.25 h, 3 h, 6 h, 12 h, 24 h, 60 h post stress treatments and quick frozen in liquid nitrogen. The expression of stress marker genes (*SOS1*, *ERF5*, *DREB2B*, *P5CS1*, *NHX1*, and *NCED*) related to biotic and abiotic stresses were also checked in 6 h treated shoot samples. To study the tissue-specific expression patterns in *B*. *rapa*, young leaves, stems and roots from one-month-old plants were collected. Apart from these tissues, the expression of *ALDH* genes was also checked in flower and siliques of *B*. *rapa* .

Unstressed plants grown under similar conditions in water without any stress were used as controls for normalization. The samples were collected as three biological and technical replicates for the expression study by qRT-PCR. *B*. *rapa ubiquitin* and *actin2* were used as reference genes for normalization of gene expression.

### RNA isolation and cDNA preparation and quantitative RT-PCR of *BrALDH*

Total RNA was isolated from control and treated samples using Tri-reagent (Takara Bio, UK) following the manufacturer’s protocol. First strand cDNA was prepared by using 2 μg of total RNA using Prime-script First Strand cDNA Synthesis Kit (Takara, Biotech, UK). The cDNA was diluted to 2.5 times (1:2.5) and 1 µl of diluted cDNA was used in a total reaction volume of 10 µl containing 10 µM of each primer, 2X Fast start SYBR green, milli-Q water for the analysis using quantitative reverse transcription PCR. The conditions for amplification included initial denaturation of at 94 °C for 2 min, followed by 40 cycles of 94 °C for 30 s, annealing temperatures ranging from 54 to 58 °C for 20 s and the extension temperature of 72 °C for 30 s. The relative fold differences for each sample were determined by normalizing Ct values (cycle threshold) of each gene to the mean expression of both *ubiquitin* and *actin2* genes and were calibrated using the using ΔΔC_T_ method (Livak and Schmittgen, 2001), which gives the relative expression of each gene. Relative expression patterns of the *BrALDH* genes in different stress conditions were represented in the form of heat maps with hierarchical clustering by using GENE-E software. Primers used in qRT-PCR are listed in Supplementary Table S4. For tissue-specific expression, statistical significance was analyzed using one way ANOVA with Duncan’s Multiple Range Test (DMRT) at P ≤ 0.05 (shown by *). Relative expressions of the respective *ALDH* genes in other tissues were calibrated by root expression of each *ALDH* genes.

### Heterologous expression and functional characterization of *BrALDH7B2* in *E*. *coli* under abiotic stress treatments

While analyzing the relative expression of *ALDH* genes, we found that the transcript level of *BrALDH7B2* gene was strongly upregulated in all the stress treatments. Hence, this gene was chosen for further analysis of its expression in imparting abiotic (NaCl, and H_2_O_2_) stress tolerance in *E*. *coli* and *yeast* heterologous systems. The coding region of *BrALDH7B2* was amplified and cloned into *E*. *coli* expression vector pET28a using *Bam*HI and *Eco*RI restriction sites. The recombinant plasmid (pET28a: *BrALDH7B2*) was confirmed through restriction enzyme digestion and sequencing. The plasmids were transformed into *E*. *coli* strain BL21 (DE3) by the standard freeze-thaw method for protein expression. The pET28a vector without insert was used as a negative control. Secondary cultures of the recombinant plasmids were allowed to grow until its OD_600_ reached 0.5. Subsequently, BL21/*BrALDH7B2* cells were induced with 1 mM of IPTG for 3 h. Cells were then harvested by centrifugation for 5 min at 4000 rpm and resuspended in lysis buffer (1XTBS) by vortexing followed by sonication. After sonication, the fusion protein was isolated from the pET28a: *BrALDH7B2* carrying cells from the supernatant using Ni-NTA Agarose (Genetix, India) following manufacturers protocol. Later, 15 µl of the loading buffer and 20 µl of each of the protein samples were boiled for 5 min before loading the gel. The samples were analyzed using 12% SDS PAGE and proteins were visualized by Coomassie blue staining and were suitably destained subsequently for the detection of the over-expressed protein.

Spot assay and growth curve analysis were performed for the BL21/*BrALDH7B2* recombinant cultures for assessing the cell viability under NaCl (400 mM and 500 mM) and H_2_O_2_ (5 mM and 6 mM) treatments according to Rampuria *et al*.^46^. The plates were incubated at 37 °C for 12 h and observations were recorded for subsequent analysis.

### Immunoblotting analysis and yeast cell survival assays with *BrALDH7B2*

During qRT-PCR, the relative expression of *BrALDH10A2* was found to be consistent during abiotic stress treatments. Hence, *BrALDH10A2* gene was used as another control in addition to empty vector control. The coding sequences of *BrALDH7B2* and *BrALDH10A2* were cloned into PYES2/NTA expression vector having URA3 as a selection marker, which can be induced by the GAL1 promoter system. The empty PYES2/NTA vector and PYES2/NTA: *BrALDH10A2* (as control) and construct PYES2/NTA: *BrALDH7B2* were introduced into oxidative stress-sensitive *Saccharomyces cerevisiae* mutant, W_3_O_3_-1-A using lithium acetate method^47^. Induction and overexpression of *BrALDH7B2* were further confirmed through immunoblot detection. For immunoblot analysis, 20 ml secondary culture of PYES2/NTA: *BrALDH7B2* yeast mutant cells induced by 2% galactose were grown to a final OD_600_ of 0.5 and the protein was extracted using TCA method^48^. The protein samples obtained were resolved on 10% SDS-PAGE and electro-blotted onto polyvinylidene difluoride (PVDF) membrane. The blot was probed using anti-His antibody (Santa Cruz Biotechnology Inc., U.S.) and was developed by using chemiluminescence detection (Clarity Max^TM^ Western ECL substrate- Bio-Rad).

Simultaneously for spot assays, secondary cultures of the W_3_O_3_-1-A yeast cells carrying PYES2/NTA, PYES2/NTA: *BrALDH10A2*, and PYES2/NTA: *BrALDH7B2* were grown for spot assays in SC-Ura medium having 2% galactose to an OD_600_ to 1. The cultures were then serially diluted to tenfold series (10^−1^ to 10^−3^) and 7 μl of the cultures were spotted on YPD plates containing 400/500 mM NaCl, or 5/6 mM H_2_O_2._ Plates were incubated for 48 h at 30 °C and cell resistance data by spot assay were recorded.

### Nuclear staining of W_3_O_3_-1-A mutant by PI treatment

To monitor the non-viable yeast cells Propidium iodide (PI) staining was used. PI is commonly used for staining the dead cells, which have lost the membrane integrity. Staining of W_3_O_3_-1-A cells carrying PYES2/NTA and PYES2/NTA: *BrALDH7B2* was performed following the protocol as described by Dalal *et al*.^49^ at 5 mM and 6 mM H_2_O_2_ concentration using 10 µg/ml PI (Sigma–Aldrich) at 30 °C for 5 min. Laser-scanning confocal fluorescence microscope (LSM 710 NLO ConfoCor 3) was used for obtaining the images. For quantification of dead cells, three fields of view from each slide were chosen randomly. More than 300 cells from three independent experiments were counted and plotted in the form of graph.

## Supplementary information

Supplementry information
