# Supplementary material for: Genome-wide characterization of ALDH Superfamily in Brassica rapa and enhancement of stress tolerance in heterologous hosts by BrALDH7B2 expression
Source: Sci Rep. 2019 May 7;9:7012. doi: 10.1038/s41598-019-43332-1 (PMC6505040; doi:10.1038/s41598-019-43332-1)
Supplement: Supplementary file 1 — Supplementry information [file 41598_2019_43332_MOESM1_ESM.pdf]

Genome-wide characterization of *ALDH* Superfamily in *Brassica rapa* and enhancement of stress tolerance in heterologous hosts by *BrALDH7B2* expression

Ranjana Gautam[1*], Israr Ahmed[1], Pawan Shukla[1,2], Rajesh Kumar Meena[1] & P.B. Kirti[1,3*]

Supplementary information

Supplementary Table S1:- Table summarizes the Protein properties of Aldehyde dehydrogenase Superfamily showing length, MW, ligands, ligand binding residues, GRAVY etc.

Protein type	Length (amino acids)	MW (kDa)	p*I*	LCR	Disordered protein (%)	Alpha helix (%)	Beta-Strand (%)	Ligands	Ligand Binding residues	GRAVY
BrALDH2C1	501	54.28	6.05	-	6	38	21	Mg	ASP108, GLN196	-0.090
BrALDH2B1	539	58.58	7.60	2-25	10	42	20	Mg	GLN234	-0.089
BrALDH2B2	534	58.36	8.71	7-24	12	40	19	Mg	GLN233	-0.135
BrALDH2B3	537	58.58	6.84	3-24	11	42	19	Mg	GLN232	-0.091
BrALDH2B4	536	58.32	7.66	1-19	10	41	19	Mg	THR75	-0.061
BrALDH3H1	301	33.44	7.19	-	5	42	19	Mg	PHE2, 244 LYS3, LYS29, SER31, GLU32, 63, THR64,78, CYS79, SER80, ILE 83,8 7, GLU101, 242 LEU102,	0.019

									GLY103, CYS148, ARG197, ILE243	
BrALDH3H2	482	53.008	7.62	-	6	46	16	Mg	PRO150	0.068
BrALDH3H3	482	53.05	7.06	-	5	47	15	Mg	PRO150	0.068
BrALDH3I1	556	60.82	8.55	-	9	44	16	-	ILE188, SER189, 217, ALA190, TRP191, ASN192, LEU197, 254, 288, 444, LYS215, PRO216, SER217, GLU218, 287, 416 GLY245, 245, 265, 266, 289, VAL247, 269, THR 250, 251, 264, CYS 322, PHE263, 484	0.001
BrALDH3F1	481	53.51	7.56	-	5	48	15	Mg	PRO145	-0.023
BrALDH3F2	484	53.89	8.29	-	6	47	15	Mg	PRO148	-0.035
BrALDH3F3	467	52.04	8.09	-	5	47	53	Mg	PRO148	-0.025
BrALDH5F1	530	56.67	7.50	20-31	8	42	19	Mg	LEU228	0.024
BrALDH6B1	527	56.61	6.62	-	9	39	20	Mg	VAL53, LYS207	-0.001
BrALDH7B1	230	24.64	8.55	-	13	30	18	Mg	MET1, GLU15, LEU16, SER17, GLY18, VAL52, GLU144, PHE146, 211 LEU170, THR217, GLU222	-0.176
BrALDH7B2	504	53.65	5.35	-	5	40	21	Mg	THR193	0.051
BrALDH10A1	503	54.80	5.84	-	6	38	19	Mg	SER265	-0.043
BrALDH10A2	501	54.50	5.35	-	6	38	20	Mg	SER265	-0.038
BrALDH11A1	496	53.15	7.01	-	5	40	21	Mg	ILE165, PRO165, 166, 193, 194, PHE168, 393, 458, ASN169, TYR170, LEU174, 265, GLY229, 245, 246, 266, ASP230,	0.021

									THR244, GLU264, 391, SER464	
BrALDH11A2	496	53.20	6.43	-	5	38	21	Mg	ILE165, PRO166,167, 193, 194, PHE168, 243, 393, ASN169, TYR170, LEU174, 265, 419, LYS192, GLY229, 245, 246, 266, ASP230, THR244, GLU264,391, CYS298, SER464	0.015
BrALDH12A1	547	60.71	6.72	-	10	41	19	Mg	VAL204, 232, 287, THR205, 282, PRO206, PHE207, 281, 444, ASN208, 268, ILE213, LYS231, 290, ASP233, 303, MET267, SER284, ARG286, GLU302, 442, ALA304, CYS337, HIS469, ALA512	-0.154
BrALDH12A2	167	18.85	6.51	-	17	69	5	1G1P, 2AMP	LEU85, PHE89	-0.260
BrALDH22A1	593	65.75	7.55	-	11	43	17	1CA 1Mg	ASP303 CYS68, HIS226	0.032

Supplementary Table S2. Table summarizes the details of *B. rapa* ALDH proteins.

Gene Name	Strand (+/-)	NCBI protein Id	Arabidopsis Orthologs (Gene ID)	Putative Molecular Function	Biological function
BrALDH2C1	negative	XP_009135901	AT3G24503	coniferyl-aldehyde dehydrogenase activity	phenylpropanoid biosynthetic process
BrALDH2B1	positive	XP_009149853	AT3G48000 AT1G23800	ATP binding, oxidoreductase activity	response to cadmium ion
BrALDH2B2	positive	XP_009150211		oxidoreductase activity	-
BrALDH2B3	positive	XP_009110081		oxidoreductase activity	-
BrALDH2B4	positive	XP009115500		-	-
BrALDH3H1	negative		AT1G44170		
BrALDH3H2	positive	XP_009107480		aldehyde dehydrogenase [NAD(P)+] activity	cellular aldehyde metabolic process
BrALDH3H3	positive	XP_009145048		aldehyde dehydrogenase [NAD(P)+] activity	cellular aldehyde metabolic process
BrALDH3I1	negative	NP_001288985	AT4G34240	aldehyde dehydrogenase (NAD) activity	cellular aldehyde metabolic process , response to abscisic acid, response to water deprivation
BrALDH3F1	negative	XP_009145598	AT4G36250	aldehyde dehydrogenase [NAD(P)+] activity	cellular aldehyde metabolic process
BrALDH3F2	positive	XP_009138415		aldehyde dehydrogenase [NAD(P)+] activity	cellular aldehyde metabolic process
BrALDH3F3	positive	XP_009109317		aldehyde dehydrogenase [NAD(P)+] activity	cellular aldehyde metabolic process
BrALDH5F1	negative	XP_009106666	AT1G79440	copper ion binding Source, NAD binding, succinate- semialdehyde dehydrogenase (NAD+) activity, succinate- semialdehyde dehydrogenase [NAD(P)+] activity	gamma-aminobutyric acid catabolic process, glutamate decarboxylation to succinate, reactive oxygen species metabolic process, response to light stimulus, response to heat
BrALDH6B1	negative	XP_009112235	AT2G14170	copper ion binding , methylmalonate- semialdehyde dehydrogenase (acylating) activity	response to oxidative stress
BrALDH7B1	positive		AT1G54100	oxidoreductase activity	NIL
BraALDH7B2	positive	XP_009106938		oxidoreductase activity	
BrALDH10A1	positive	XP_009150209.1	AT3G48170 AT1G74920	betaine-aldehyde dehydrogenase activity	response to abscisic acid, response to water deprivation
BrALDH10A2	positive	XP_009104738		oxidoreductase activity Biological function-	response to salt stress, response to water deprivation
BrALDH11A1	positive	XP_009117140	AT2G24270	oxidoreductase activity	
BrALDH11A2	negative	XP_0091404530		oxidoreductase activity	
BrALDH12A1	negative	XP_009151373	AT5G62530	1-pyrroline-5-carboxylate dehydrogenase activity, cobalt ion binding, zinc ion binding	proline catabolic process to glutamate, reactive oxygen species metabolic process, response to salt stress
BrALDH12A2	negative			oxidoreductase activity	-
BrALDH22A1	positive	XP_009147139	AT3G66658	oxidoreductase activity	-

Supplementary Table S3: Motif enrichment analysis using CentriMo enrichment Analysis tool by MEME in *ALDH* genes.

consensus-Motif	E-value	Fischer E-value	Adjusted p value	Region width	Total sites	p-value	Negative p value
TTGACC	4.50E+00	1.00E+00	9.00E-01	259	23	4.70E-03	1.00E+00
ATTTCAAA	5.00E+00	1.00E+00	1.00E+00	11	23	2.70E-02	1.00E+00
TGACG	5.00E+00	1.00E+00	1.00E+00	2	21	1.00E+00	1.00E+00
CAACTG	5.00E+00	1.00E+00	1.00E+00	1	23	1.00E+00	1.00E+00
AAAAAATTTC	5.00E+00	1.00E+00	1.00E+00	1	23	1.00E+00	1.00E+00
AACGAC	7.00E+00	1.00E+00	1.00E+00	1	23	1.00E+00	1.00E+00
GAGAAGAATA	7.00E+00	1.00E+00	1.00E+00	1	18	1.00E+00	1.00E+00
ATTTTCTTCA	7.00E+00	1.00E+00	1.00E+00	1	22	1.00E+00	1.00E+00
CGTCA	7.00E+00	1.00E+00	1.00E+00	2	21	1.00E+00	1.00E+00
TACGTG	7.00E+00	1.00E+00	1.00E+00	1	22	8.70E-02	1.00E+00
GTTTTCTTAC	7.00E+00	1.00E+00	1.00E+00	1	20	1.00E+00	1.00E+00
CAGAAAAGGA	7.00E+00	1.00E+00	1.00E+00	1	18	1.00E+00	1.00E+00
CCGAAA	5.90E+00	9.90E-01	8.40E-01	99	23	3.70E-03	1.00E+00
CACGTG	7.00E+00	1.00E+00	1.00E+00	1	20	2.00E-02	1.00E+00
ACGTGGC	7.00E+00	1.00E+00	1.00E+00	4	11	4.30E-02	1.00E+00
TGACGTAA	7.00E+00	1.00E+00	1.00E+00	1	20	1.00E+00	1.00E+00
CCATCTTTTT	7.00E+00	1.00E+00	1.00E+00	1	23	1.00E+00	1.00E+00
CCTTTTG	7.00E+00	1.00E+00	1.00E+00	2	22	1.00E+00	1.00E+00
TAACTG	7.00E+00	1.00E+00	1.00E+00	1	21	1.00E+00	1.00E+00
TTGACC	4.50E+00	1.00E+00	9.00E-01	259	23	4.70E-03	1.00E+00
ATTTCAAA	5.00E+00	1.00E+00	1.00E+00	11	23	2.70E-02	1.00E+00
TGACG	5.00E+00	1.00E+00	1.00E+00	2	21	1.00E+00	1.00E+00
CAACTG	5.00E+00	1.00E+00	1.00E+00	1	23	1.00E+00	1.00E+00
AAAAAATTTC	5.00E+00	1.00E+00	1.00E+00	1	23	1.00E+00	1.00E+00
AACGAC	7.00E+00	1.00E+00	1.00E+00	1	23	1.00E+00	1.00E+00
GAGAAGAATA	7.00E+00	1.00E+00	1.00E+00	1	18	1.00E+00	1.00E+00
ATTTTCTTCA	7.00E+00	1.00E+00	1.00E+00	1	22	1.00E+00	1.00E+00
CGTCA	7.00E+00	1.00E+00	1.00E+00	2	21	1.00E+00	1.00E+00
TACGTG	7.00E+00	1.00E+00	1.00E+00	1	22	8.70E-02	1.00E+00
GTTTTCTTAC	7.00E+00	1.00E+00	1.00E+00	1	20	1.00E+00	1.00E+00
CAGAAAAGGA	7.00E+00	1.00E+00	1.00E+00	1	18	1.00E+00	1.00E+00

CentriMo (Local Motif Enrichment Analysis): Version 5.0.5 compiled on Mar 19 2019 at 18:54:06.
The format of this file is described at http://meme-suite.org//doc/centrimo-output-format.html.
centrimo --oc . --verbosity 1 --score 5.0 --ethresh 10.0 --bfile sequences.fa.bg --neg comparative_sequences.fa sequences.fa motifs.meme.

Supplementary Table S4: Table showing primer details used in real-time experiment.

**BrALDH10A1F**	ACTGAGGATGAGGCAATACAGC
**BrALDH10A1R**	TGACCCACACAATACCCGC
**BrALDH10A2F**	CTCACAGCCTTGCTTTACTCA
**BrALDH10A2R**	AGAGCGTCACTTGCTTCACA
**BrALDH11A1F**	ACCGTTTGGTCCTGTCTTACC
**BrALDH11A1R**	TGCCTTGTTGATGTCCTTTGT
**BrALDH11A2F**	GTCCTGTCTTGCCTGTCTTGA
**BrALDH11A2R**	CCGTTCCTGTCTCCATTGC
**BrALDH12A2F**	ATGCTCGCCTTACCAAAGGT
**BrALDH12A2R**	TGCATTACCTGATCGCCACA
**BrALDH12A1F**	ACTCGGTGAATGGGACTACAT
**BrALDH12A1R**	ACTTTATTGCCTCTGGTGTTCC
**BrALDH122A1F**	CAGCGTCGTGCCAAACAAA
**BrALDH122A1R**	CCGTCCAAACCCACTATCCT
**BrALDH2C1F**	TGCTCTTGATGCGGTTGACG
**BrALDH2C1R**	GAAGTGTCTCGCCGTGGATT
**BrALDH2B1F**	TTTTCGGTCCAGTCCAGTCC
**BrALDH2B1**	GCAGTGTCCAGGCTCTTTGT
**BrALDH2B2F**	TGCTCTCTTCTTCAACCAGGG
**BrALDH2B2R**	GCGTGCCTTTGCTTTCTCAA
**BrALDH2B3F**	CGGACCAGTTCAGACCATACTT
**BrALDH2B3R**	CGCTCGCATCAGCCTATTC
**BrALDH2B4F**	ATGTGGATCAGGCCGTTGAG
**BrALDH2B4R**	GCGTTTGATTGCACGAGCTT
**BrALDH3H1F**	GCTTCTTCTTCTCTGCTCGC
**BrALDH3H1R**	ACGCCCAATTCTCGAACTACA
**BrALDH3H2F**	CGTCTATGGGGGTCAGAAGAA
**BrALDH3H2R**	GTTGTTGAGCGTGAGGATTGG
**BrALDH3H3F**	TCCTTCCAATCATCACGCTCA
**BrALDH3H3R**	GCCTCCAGCAGAAACTGTCA
**BrALDH3I1F**	TGGCAGCGTATCTCTTCACAG
**BrALDH3I1R**	CCCAACTCCTCCAAACGGTA
**BrALDH3F1F**	GCATTCACCAAGGACGAGAA
**BrALDH3F1R**	AAGGGCAACGCATCACACAT
**BrALDH3F2F**	CATTCACCAAGGACGAGAAACT
**BrALDH3F2R**	AAAGGGCAACGCATCACACA
**BrALDH3F3F**	ACCAAAGCCACTTGCCATCT
**BrALDH3F3R**	CTGTCCTACTCCTCCAAAGGG
**BrALDH5F1F**	TTCAAAACTGAGGAGGACGCT
**BrALDH5F1R**	CTTCGGATACACGCCACGA
**BrALDH6B1F**	GATGTTTCCCATTGCGGTGA
**BrALDH6B1R**	GAACCCCGTCAGGTAATCCA
**BrALDH7B2F**	TCCTCTTGGCATTGTCGGTG
**BrALDH7B1R**	TCCAGACTACACAGTTTCCGC
**BrALDH7B1F**	TTGTGGAGCCTACGATACTTG
**BrALDH7B1R**	TCGCTACTTGCTTCTTCAAATG
**NHX1F**	AGCAGCATTCGTCTTCCCAT
**NHX1R**	TGTGCCCAGCACTTGTAAAC

**P5CS1F**	GCAGAATGGTGTGCTAAACGAG
**P5CS1R**	TACAATTTCAACGGTTCAGGC
**SOS1F**	AGTTTCTCAAGACAAGCAACACA
**SOS1R**	ATGATCTCTGGAGCTGGTGC
**NCEDF**	TCGTCGTTTTACCGGACCAG
**NCEDR**	AGGTGGAAAGCAGAAGCAGTC
**DREB2B F**	CCGTTGCGGATTATGGTTGG
**DREB2B R**	TCCCGTTCTGGTCTTCATCC
**ERF5 F**	TCTAACCGAAACCCGCCTTC
**ERF5 R**	TTTCCCCCACGGTCTTTGTC
**SOS1 F**	AGTTTCTCAAGACAAGCAACACA
**SOS1 R**	ATGATCTCTGGAGCTGGTGC

Supplementary Table S5: List of upregulated and downregulated genes in shoots during abiotic and hormonal treatments at different time intervals. ↑- represents upregulation, ↓- downregulation and ↔ - no change in transcript level.

	NaCl (100 mM)						PEG (10%)						H_2O_2 (10 mM)						ETH (1% v/v)						ABA (100 µM)					
**Gene name**	**0.25 h**	**3 h**	**6 h**	**12 h**	**24 h**	**60 h**	**0.25 h**	**3 h**	**6 h**	**12 h**	**24 h**	**60 h**	**0.25 h**	**3 h**	**6 h**	**12 h**	**24 h**	**60 h**	**0.25 h**	**3 h**	**6 h**	**12 h**	**24 h**	**60 h**	**0.25 h**	**3 h**	**6 h**	**12 h**	**24 h**	**60 h**
BrALDH2B1	↓	↑	↑	↑	↑	↑	↓	↑	↑	↑	↔	↓	↓	↔	↑	↑	↑	↑	↔	↑	↑	↑	↑	↑	↓	↓	↓	↔	↓	↑
BrALDH2B2	↔	↑	↑	↑	↑	↑	↔	↑	↑	↔	↔	↔	↑	↔	↑	↑	↑	↑	↑	↔	↔	↑	↔	↑	↑	↑	↑	↔	↔	↑
BrALDH2B3	↔	↑	↑	↑	↑	↑	↔	↑	↑	↔	↔	↓	↑	↑	↑	↑	↑	↑	↑	↑	↑	↑	↑	↔	↑	↑	↑	↓	↓	↑
BrALDH2B4	↓	↑	↑	↑	↑	↑	↑	↑	↑	↔	↓	↓	↑	↓	↓	↑	↓	↔	↑	↑	↑	↓	↑	↔	↑	↔	↑	↑	↓	↑
BrALDH2C1	↑	↓	↓	↓	↓	↓	↑	↓	↓	↓	↓	↓	↑	↑	↑	↔	↑	↔	↑	↓	↓	↔	↓	↔	↑	↔	↓	↓	↓	↔
BrALDH3H1	↓	↔	↓	↔	↔	↓	↓	↑	↔	↓	↔	↓	↓	↓	↓	↓	↓	↓	↓	↓	↔	↔	↓	↓	↓	↓	↓	↓	↓	↓
BrALDH3H2	↓	↑	↑	↑	↑	↓	↓	↑	↑	↔	↔	↓	↓	↓	↓	↓	↓	↓	↓	↔	↔	↑	↔	↓	↓	↑	↑	↔	↔	↔
BrALDH3H3	↑	↑	↑	↑	↑	↑	↑	↑	↑	↔	↔	↔	↑	↔	↑	↔	↑	↑	↑	↔	↔	↑	↔	↑	↑	↑	↑	↔	↔	↑
BrALDH3I1	↓	↓	↓	↔	↑	↓	↓	↓	↓	↓	↓	↓	↑	↑	↑	↓	↑	↓	↑	↓	↔	↔	↓	↓	↑	↓	↓	↓	↓	↓
BrALDH3F1	↔	↑	↔	↔	↑	↑	↔	↑	↔	↓	↔	↓	↔	↔	↔	↔	↓	↔	↔	↔	↔	↔	↔	↓	↑	↔	↔	↔	↔	↔
BrALDH3F2	↓	↑	↓	↑	↑	↔	↓	↓	↓	↑	↓	↓	↔	↔	↔	↓	↑	↓	↔	↓	↔	↓	↔	↓	↑	↔	↔	↔	↓	↓
BrALDH3F3	↓	↑	↔	↑	↑	↑	↔	↔	↑	↔	↑	↔	↔	↔	↔	↔	↓	↓	↔	↔	↔	↔	↔	↓	↑	↑	↔	↔	↔	↔
BrALDH5F1	↔	↑	↑	↑	↑	↓	↔	↑	↑	↑	↑	↑	↑	↓	↑	↑	↔	↑	↑	↑	↑	↑	↑	↑	↑	↑	↑	↑	↑	↑
BrALDH6B1	↓	↔	↓	↑	↑	↑	↓	↓	↔	↔	↓	↓	↓	↓	↔	↔	↔	↑	↔	↔	↔	↑	↑	↔	↔	↔	↔	↑	↔	↔
BrALDH7B1	↓	↔	↓	↑	↑	↑	↓	↑	↔	↑	↓	↓	↓	↑	↑	↔	↔	↔	↓	↑	↔	↑	↑	↓	↓	↑	↑	↑	↓	↓
BrALDH7B2	↑	↑	↑	↑	↑	↑	↑	↑	↑	↑	↔	↑	↑	↑	↑	↑	↑	↑	↑	↑	↑	↑	↑	↑	↑	↑	↑	↑	↑	↑
BrALDH10A1	↔	↔	↓	↑	↑	↑	↔	↔	↔	↓	↓	↔	↑	↔	↔	↑	↔	↑	↑	↔	↔	↑	↔	↔	↑	↔	↔	↔	↔	↑
BrALDH10A2	↔	↔	↔	↔	↔	↔	↔	↔	↔	↔	↔	↔	↔	↔	↔	↔	↔	↔	↑	↓	↔	↔	↔	↔	↑	↔	↔	↓	↔	↑
BrALDH11A1	↔	↓	↓	↓	↔	↑	↔		↓	↓	↓	↓	↑	↓	↓	↓	↓	↓	↑	↓	↓	↓	↔	↓	↑	↓	↓	↓	↓	↔
BrALDH11A2	↔	↓	↔	↓	↔	↑	↔	↓	↓	↔	↓	↓	↑	↓	↓	↓	↓	↓	↑	↓	↓	↓	↓	↓	↑	↓	↓	↓	↓	↑
BrALDH12A1	↔	↔	↑	↔	↑	↑	↔	↑	↑	↔	↑	↑	↑	↔	↔	↔	↔	↔	↑	↔	↑	↑	↑	↑	↑	↑	↑	↑	↑	↑
BrALDH12A2	↓	↓	↑	↔	↑	↓	↓	↓	↓	↓	↓	↓	↑	↔	↑	↑	↓	↑	↑	↓	↔	↑	↓	↔	↑	↔	↔	↔	↔	↓
BrALDH22A1	↑	↑	↑	↑	↑	↑	↔	↔	↔	↔	↔	↔	↑	↑	↑	↑	↑	↑	↑	↔	↑	↓	↑	↔	↑	↓	↓	↑	↓	↑

Supplementary Table S6: List of upregulated and downregulated genes in roots during abiotic and hormonal treatments at different time intervals. ↑- represents upregulation, ↓- downregulation and ↔ - no change in transcript level.

	NaCl (100 mM)						PEG (10%)						H_2O_2 (10 mM)						ETH (1% v/v)						ABA (100 µM)					
**Gene name**	**0.25 h**	**3 h**	**6 h**	**12 h**	**24 h**	**60 h**	**0.25 h**	**3 h**	**6 h**	**12 h**	**24 h**	**60 h**	**0.25 h**	**3 h**	**6 h**	**12 h**	**24 h**	**60 h**	**0.25 h**	**3 h**	**6 h**	**12 h**	**24 h**	**60 h**	**0.25 h**	**3 h**	**6 h**	**12 h**	**24 h**	**60 h**
BrALDH2B1	↔	↓	↔	↔	↔	↑	↔	↓	↓	↓	↓	↑	↔	↓	↓	↓	↓	↑	↔	↓	↓	↓	↓	↑	↔	↓	↓	↓	↓	↑
BrALDH2B2	↔	↓	↓	↓	↓	↑	↔	↓	↓	↓	↓	↑	↔	↓	↓	↓	↓	↓	↔	↓	↓	↓	↓	↓	↔	↓	↓	↓	↓	↑
BrALDH2B3	↑	↔	↑	↑	↑	↑	↔	↓	↓	↓	↓	↑	↑	↓	↓	↓	↓	↑	↑	↓	↓	↓	↓	↑	↑	↓	↓	↓	↓	↔
BrALDH2B4	↑	↑	↓	↓	↓	↑	↓	↓	↔	↓	↓	↑	↑	↔	↓	↓	↓	↑	↔	↔	↓	↔	↓	↑	↑	↔	↑	↔	↔	↔
BrALDH2C1	↔	↔	↑	↔	↔	↑	↔	↑	↑	↑	↑	↓	↓	↑	↑	↑	↑	↑	↓	↑	↑	↑	↔	↑	↔	↑	↑	↑	↑	↑
BrALDH3H1	↔	↑	↔	↔	↔	↔	↔	↓	↓	↓	↓	↓	↓	↓	↓	↓	↓	↔	↓	↓	↓	↓	↓	↑	↓	↓	↓	↓	↓	↓
BrALDH3H2	↔	↔	↔	↔	↔	↔	↓	↓	↔	↔	↔	↓	↓	↔	↔	↔	↔	↔	↓	↓	↔	↔	↔	↓	↓	↔	↑	↔	↔	↓
BrALDH3H3	↑	↔	↔	↔	↔	↑	↔	↓	↓	↓	↓	↑	↑	↓	↓	↓	↓	↑	↔	↓	↓	↓	↓	↓	↑	↓	↓	↓	↓	↑
BrALDH3I1	↑	↑	↑	↑	↔	↔	↓	↑	↑	↑	↔	↔	↔	↑	↑	↑	↑	↔	↔	↑	↑	↔	↓	↑	↔	↑	↑	↑	↓	↓
BrALDH3F1	↓	↔	↔	↔	↔	↔	↓	↓	↓	↓	↓	↔	↓	↓	↓	↓	↓	↔	↓	↓	↓	↓	↓	↔	↓	↓	↓	↓	↓	↓
BrALDH3F2	↓	↓	↓	↓	↓	↓	↓	↓	↓	↓	↓	↔	↓	↓	↓	↓	↓	↓	↓	↓	↓	↓	↓	↔	↓	↓	↓	↓	↓	↓
BrALDH3F3	↓	↓	↓	↓	↓	↔	↓	↓	↓	↓	↓	↔	↓	↓	↓	↓	↓	↔	↓	↓	↓	↓	↓	↔	↓	↓	↓	↓	↓	↔
BrALDH5F1	↑	↓	↓	↓	↓	↑	↑	↓	↔	↔	↔	↑	↔	↔	↔	↔	↔	↑	↑	↓	↔	↑	↔	↑	↑	↔	↑	↔	↔	↑
BrALDH6B1	↔	↔	↔	↔	↔	↔	↔	↓	↔	↔	↔	↔	↔	↔	↔	↔	↔	↓	↓	↓	↔	↔	↔	↔	↓	↔	↓	↓	↓	↔
BrALDH7B1	↓	↓	↓	↓	↓	↓	↓	↓	↓	↓	↓	↓	↓	↓	↓	↓	↓	↓	↓	↓	↓	↓	↓	↑	↓	↓	↓	↓	↓	↔
BrALDH7B2	↑	↑	↑	↑	↑	↑	↔	↑	↑	↑	↑	↑	↑	↑	↑	↑	↑	↑	↔	↑	↑	↑	↑	↑	↑	↑	↑	↑	↑	↑
BrALDH10A1	↑	↔	↑	↑	↑	↑	↔	↔	↑	↑	↔	↑	↔	↑	↑	↓	↔	↔	↔	↑	↑	↑	↑	↑	↑	↑	↑	↑	↔	↔
BrALDH10A2	↔	↔	↔	↔	↔	↔	↔	↔	↔	↔	↔	↔	↔	↔	↔	↔	↔	↑	↔	↓	↓	↓	↓	↑	↓	↓	↓	↓	↓	↔
BrALDH11A1	↓	↓	↓	↓	↓	↔	↓	↔	↑	↑	↑	↔	↓	↑	↑	↑	↑	↔	↓	↑	↑	↑	↑	↑	↓	↑	↑	↓	↓	↔
BrALDH11A2	↓	↓	↓	↓	↓	↓	↓	↓	↓	↓	↓	↓	↓	↓	↓	↓	↓	↓	↓	↓	↓	↓	↔	↑	↓	↓	↓	↓	↓	↔
BrALDH12A1	↑	↓	↓	↓	↓	↑	↑	↑	↑	↑	↔	↑	↑	↑	↑	↑	↓	↑	↔	↑	↑	↑	↑	↑	↑	↑		↑	↔	↑
BrALDH12A2	↑	↓	↓	↓	↓	↑	↓	↓	↓	↓	↓	↑	↓	↓	↓	↓	↓	↑	↔	↓	↓	↓	↓	↑	↔	↓	↓	↓	↓	↓
BrALDH22A1	↑	↓	↓	↓	↓	↑	↓	↓	↓	↓	↓	↑	↔	↔	↔	↔	↔	↑	↔	↔	↔	↔	↔	↑	↔	↔	↔	↔	↔	↔

Supplementary Fig. S1:- Phylogenetic relationship constructed on MEGA7 platform by using NJ method a) between *Brassica rapa, Arabidopsis* and *Oryzae sativa ALDH*. b) Among *B. rapa.*

Supplementary Fig. S2:- Predicted three dimensional structure and ligand binding sites of all 23 identified ALDH proteins using Phyre2 and 3D ligand sites respectively.

Supplementary Fig. S3:- In-silico analysis of *B. rapa ALDH*s promoter sequences carrying stress and hormonal signal-responsive elements using nucleotide sequence >1 kb upstream of the gene. Each element is represented with a different shape and color which is explained at the bottom of the figure.

Supplementary Fig. S4:- Diagrammatic representation of the the CentriMo plots showing the distribution of the given motifs related to various stress treatments in *ALDH* genes compared to two hundred four gene of *B. rapa* not involved in stress. Dotted lines represent the distribution of motif in negative datasets whereas unbroken curve represent primary dataset provided. The p value of the identified motifs in the ALDH genes and control sequences are shown in each plot.

Supplementary Fig. S5:- Diagrammatic representation of overlap in the upregulated genes at different time intervals under abiotic and hormonal treatments; a) Shoot and b) Root.

Supplementary Fig. S6:- Diagrammatic representation of overlap in the downregulated genes at different time intervals under abiotic and hormonal treatments; a) Shoot and b) Root.

Supplementary Fig. S7:- Quantitative RT-PCR expression analysis of stress marker genes in control and shoot NaCl, H_2O_2, PEG, ABA and ethephon treated shoot samples at 6 hr in *B. rapa*. *SOS1*, *ERF5*, *DREB2B*, *P5CS1*, *NHX1* AND *NCED* marker genes were normalized in shoot using actin2 and EF1α as the reference gene. * represents statistical significance at $P \leq 0.05$ using one way ANOVA with Duncan's Multiple Range Test (DMRT). Function of stress marker genes- *SOS1*, *P5CS1* and *NHX1*- salt stress, *ERF5*- ethylene stress, *DREB2B*- drought stress, *NCED*- ABA marker.

Supplementary Fig. S8:- Cloning of *BrALDH7B2* and *BrALDH10A2* gene into pYES2/NTA; a) Full gene amplification of *BrALDH7B2 gene*; b and d) Confirmation of *BrALDH7B2* and *BrALDH10A2* through restriction digestion (release fragment 1.5kb and 1.53) from pTZ57R/T; c and e) restriction digestion through pYES2/NTA vector;

Supplementary Fig. S9: a) 12% SDS-PAGE of pET28a and *BrALDH7B2*: pET28a stained with coomassie brilliant blue G 250. Lane 1- Protein marker; Lane 2 – Control (E. coli BL21 with pET28a: *BrALDH7B2*), Lane 3- E. coli *BL21* with cell lysate induced with IPTG; Lane 4: pET28a: *BrALDH7B2* protein supernatant; b) Schematic diagram of PYES2/NTA: *BrALDH7B2* immunoblot analysis using anti-His antibodies construct used for His-tagged recombinant protein of ~55 kDa expression at 2hr and 3hr post 2% galactose. Lane 1- Protein marker; Lane 2 – 3 hr induced fusion protein *BrALDH7B2* with His-tag of ~55 kDa by 2% galactose, Lane 3- 2 hr induced *BrALDH7B2* protein and Lane 4- Control (pYESNT/A); c) Comparison of cell survival assays of *S. cerevisiae* mutant W_3O_3-1-A expressing PYES2/NTA: *BrALDH10A2* and PYES2/NTA: *BraALDH7B2* under different abiotic stress treatments up to 10^{-3} fold dilutions. W_3O_3-1-A cells at OD600 = 0.5, were spotted on YPDA medium (Yeast potato dextrose agar) supplemented with 400, 500 mM NaCl and 5, 6 mM H_2O_2 and 2% galatose.

Supplementary Fig. S1.

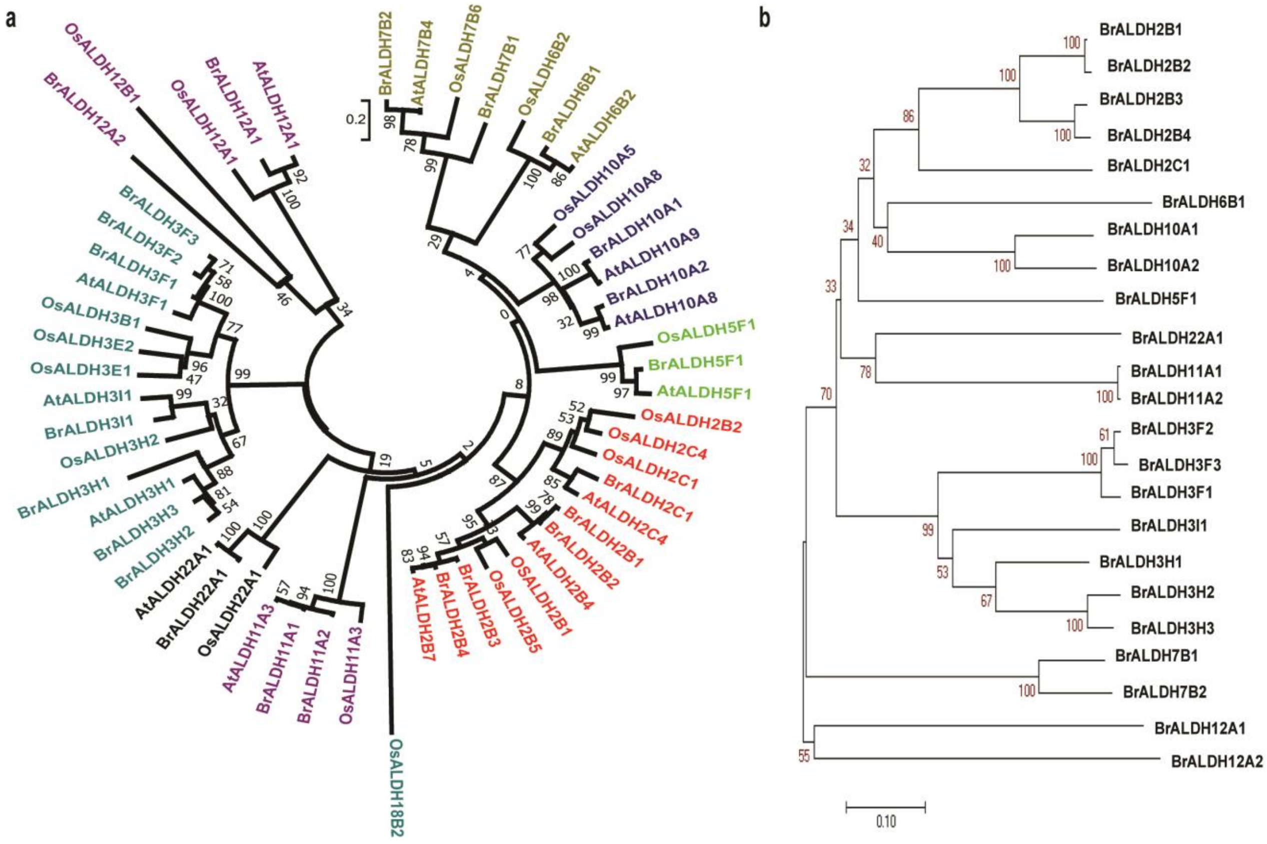

Supplementary Fig. S2.

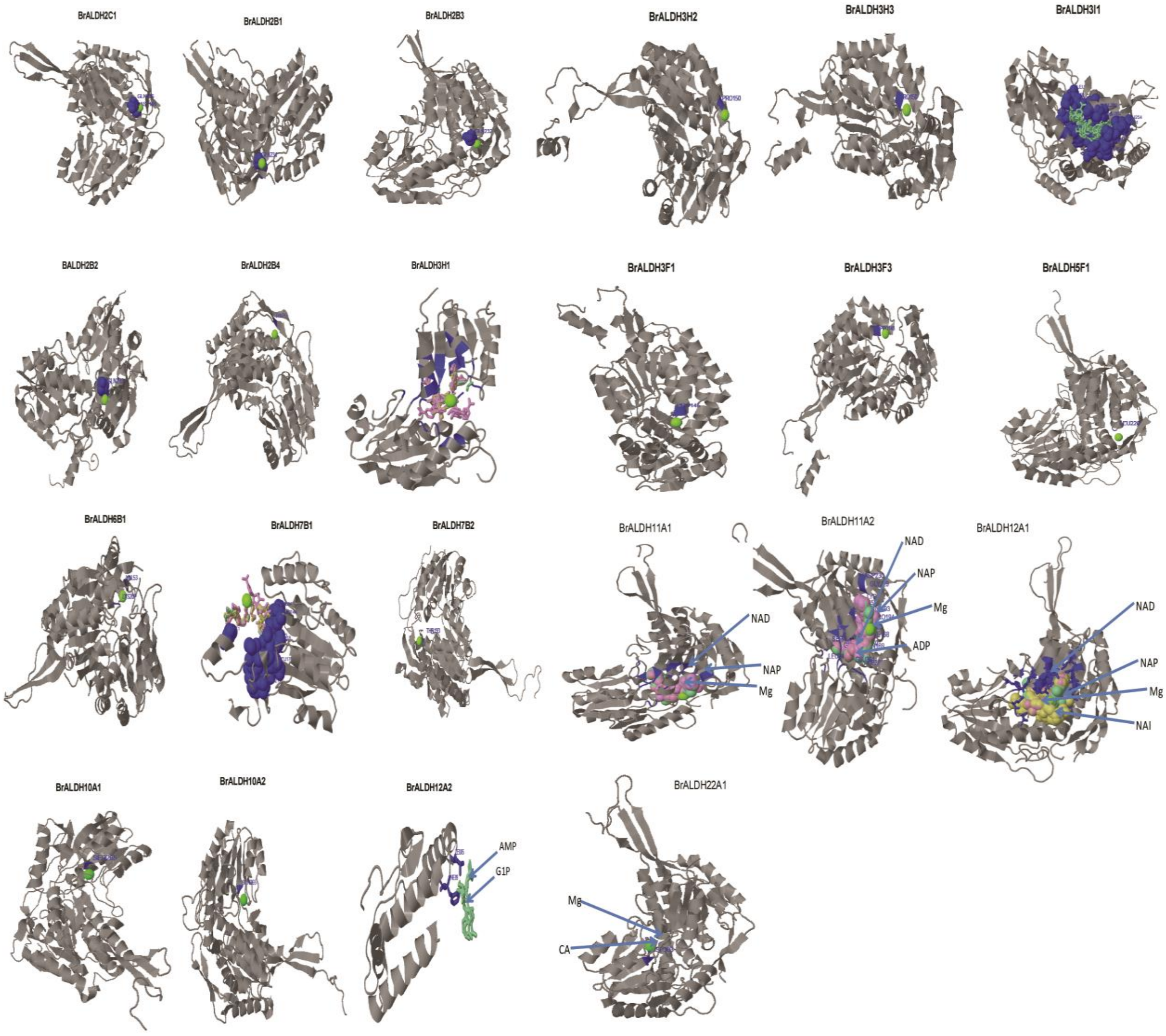

Supplementary Fig. S3.

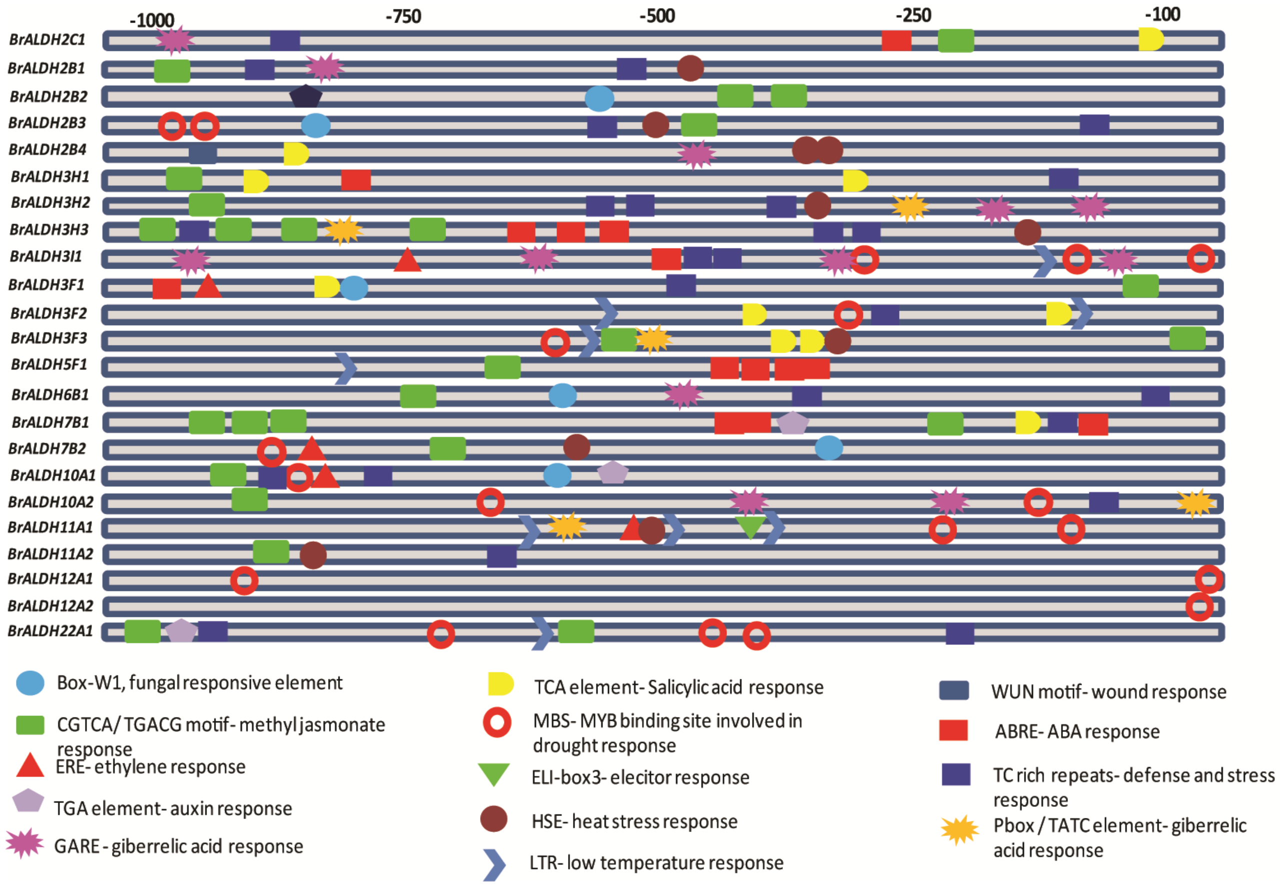

Supplementary Fig. S4.

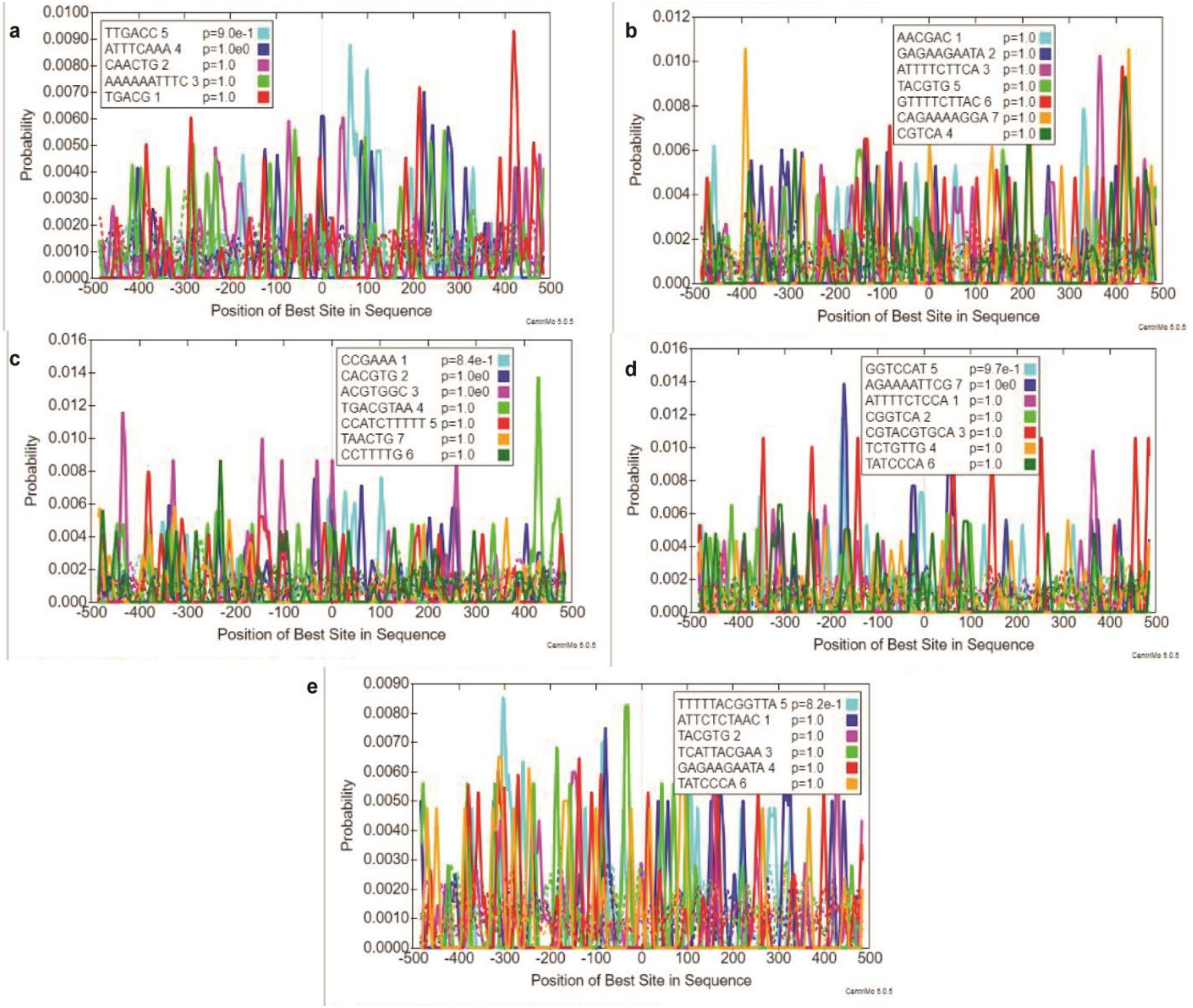

Supplementary Fig. S5.

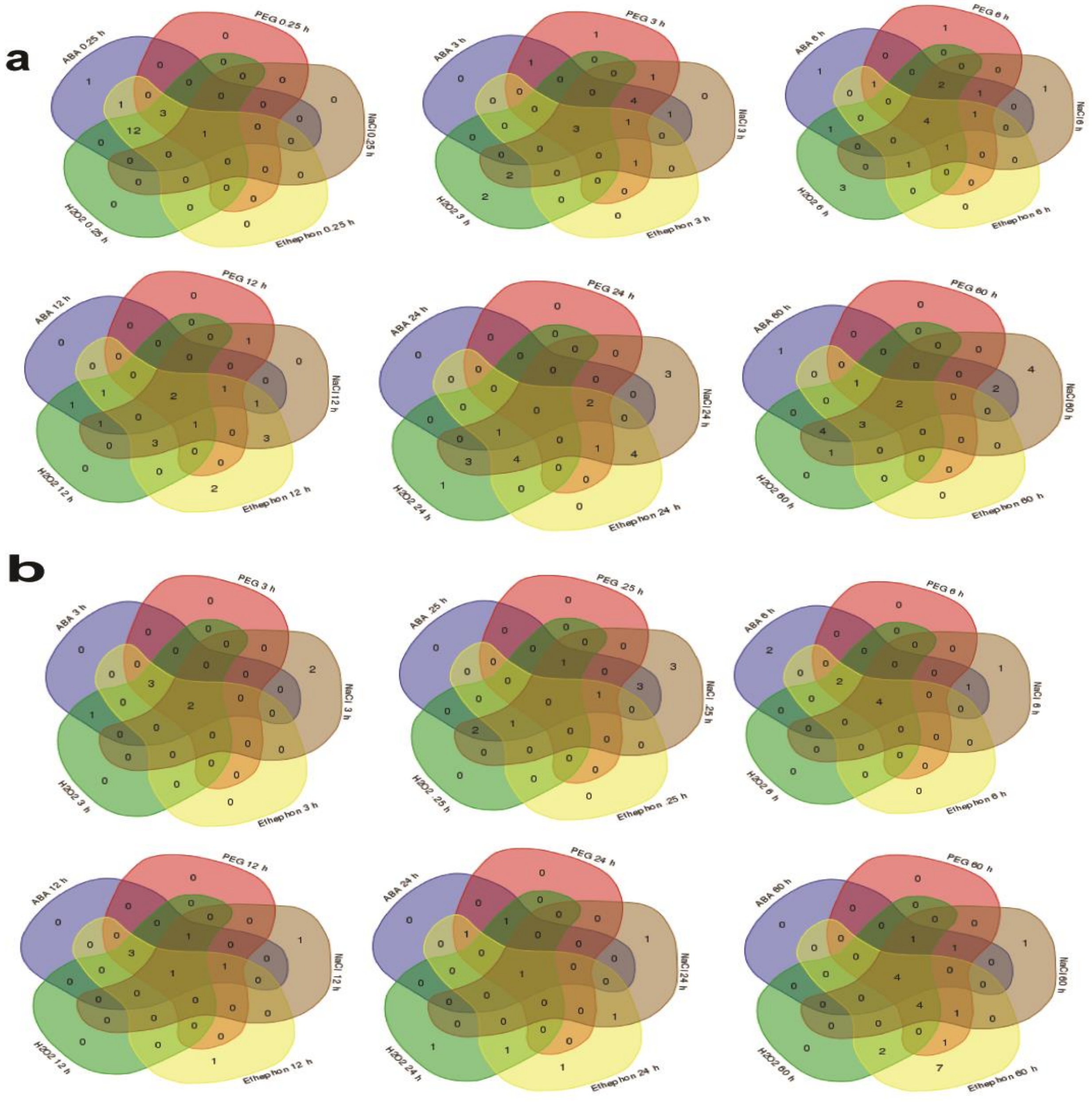

Supplementary Fig. S6.

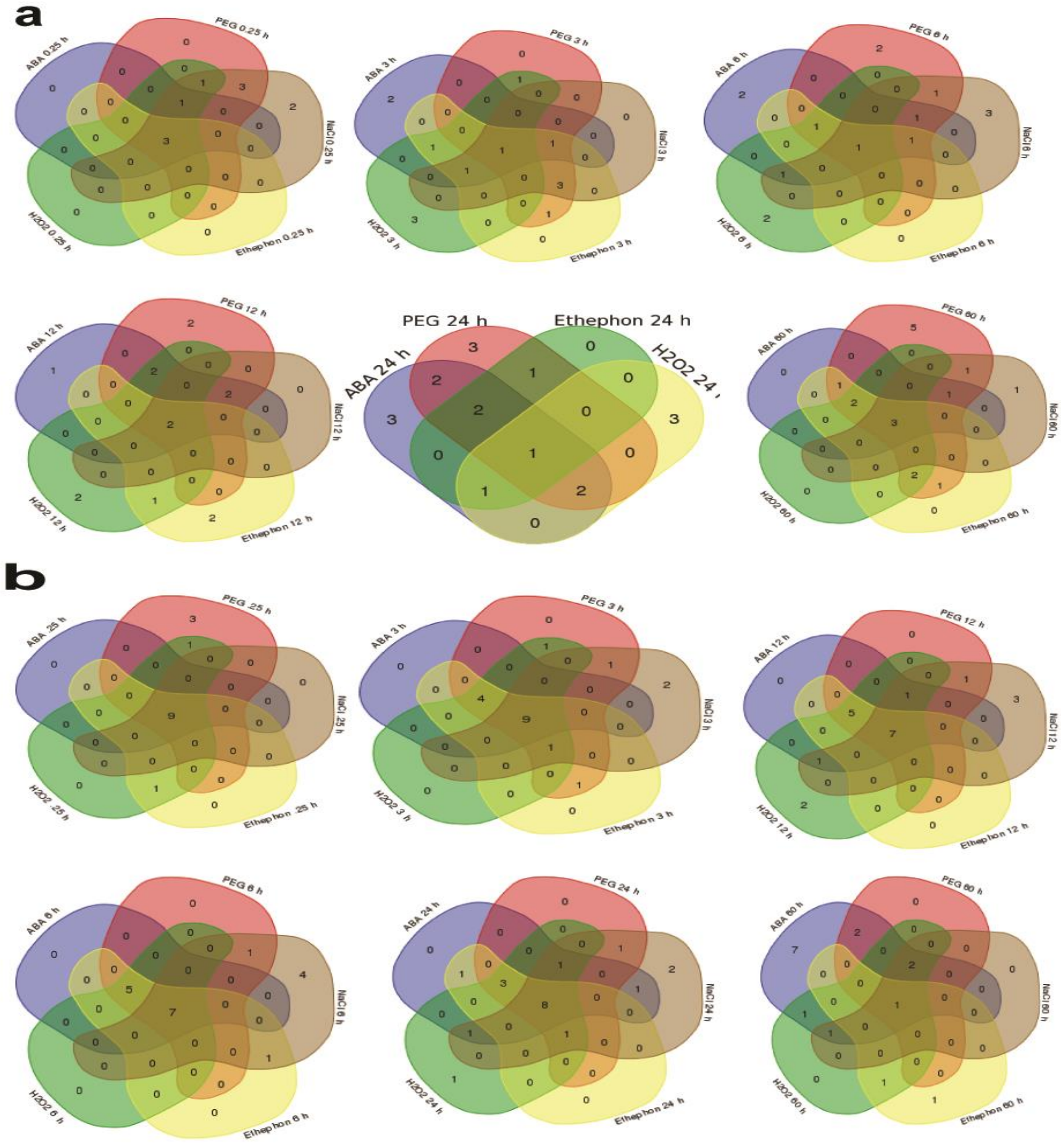

Supplementary Fig. S7.

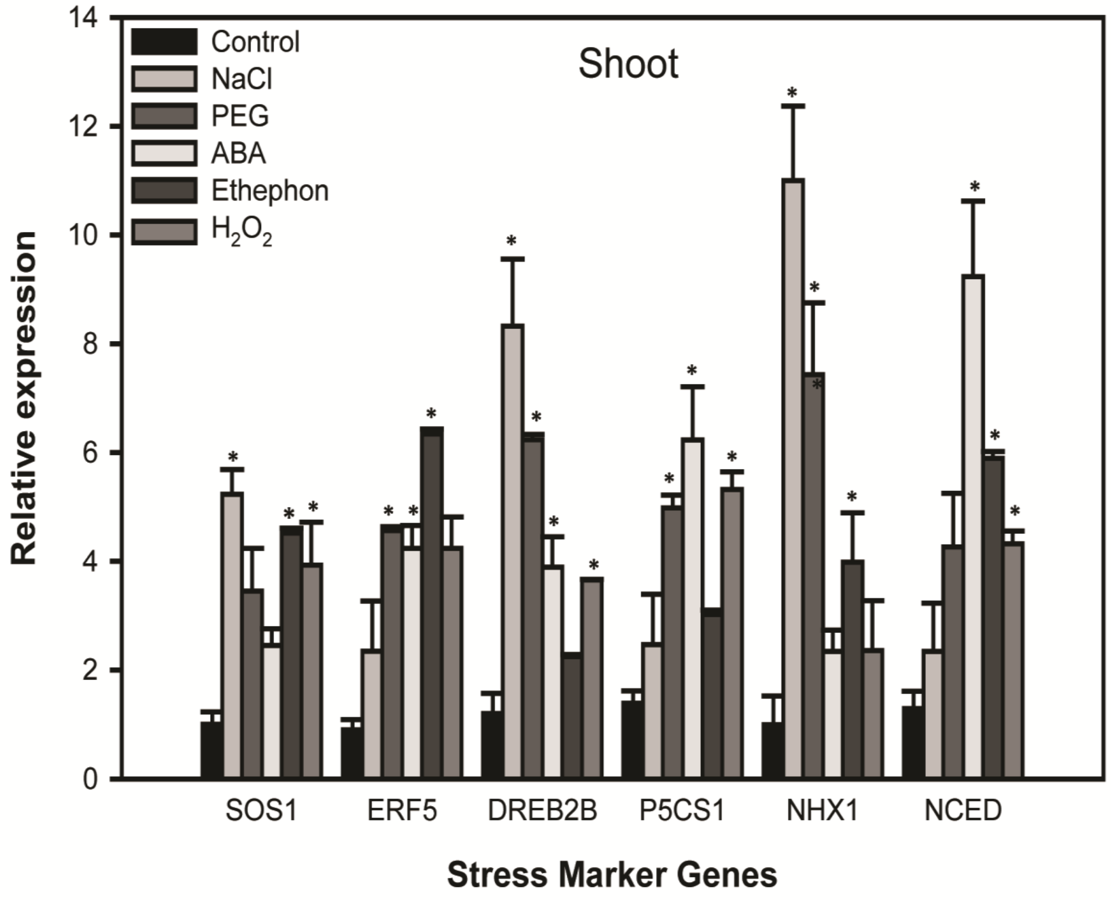

Supplementary Fig. S8.

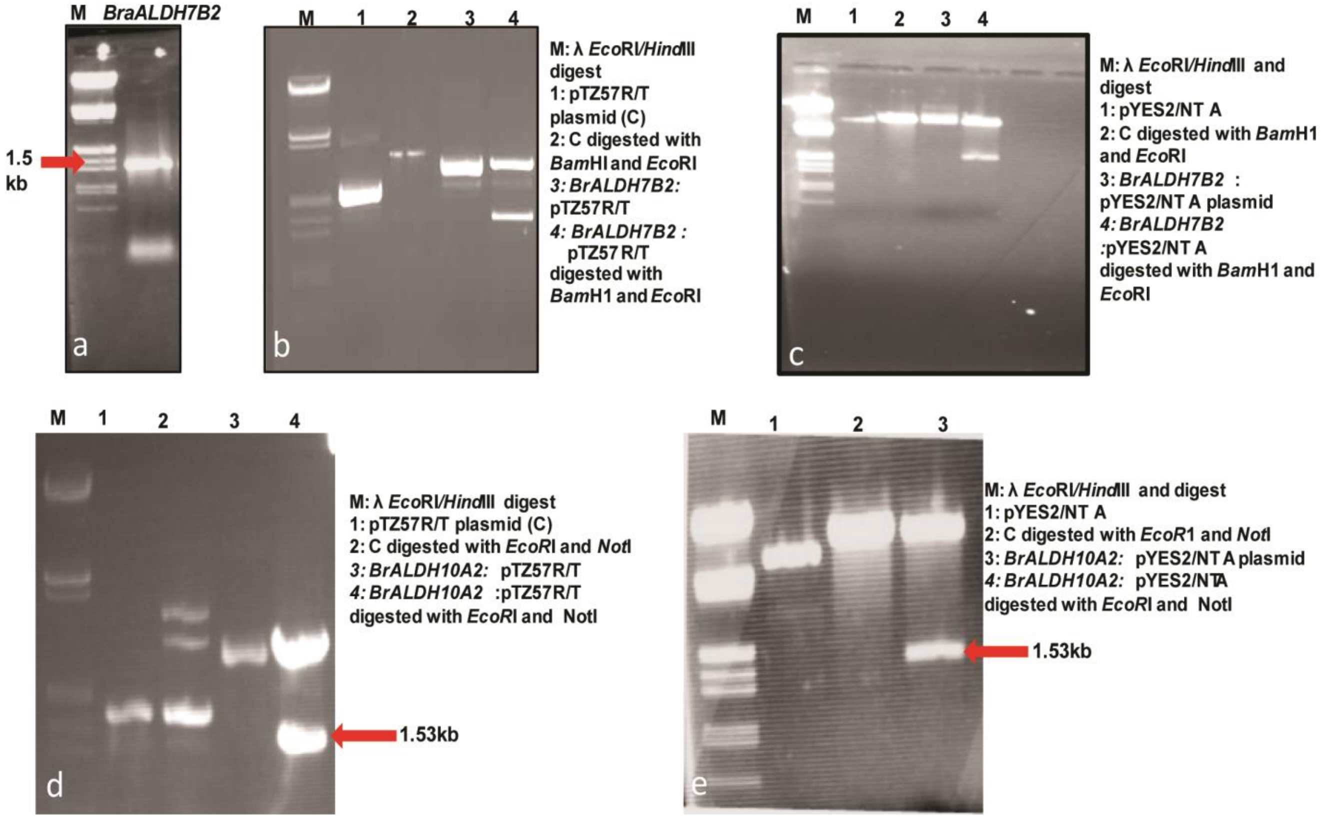

Supplementary Fig. S9.

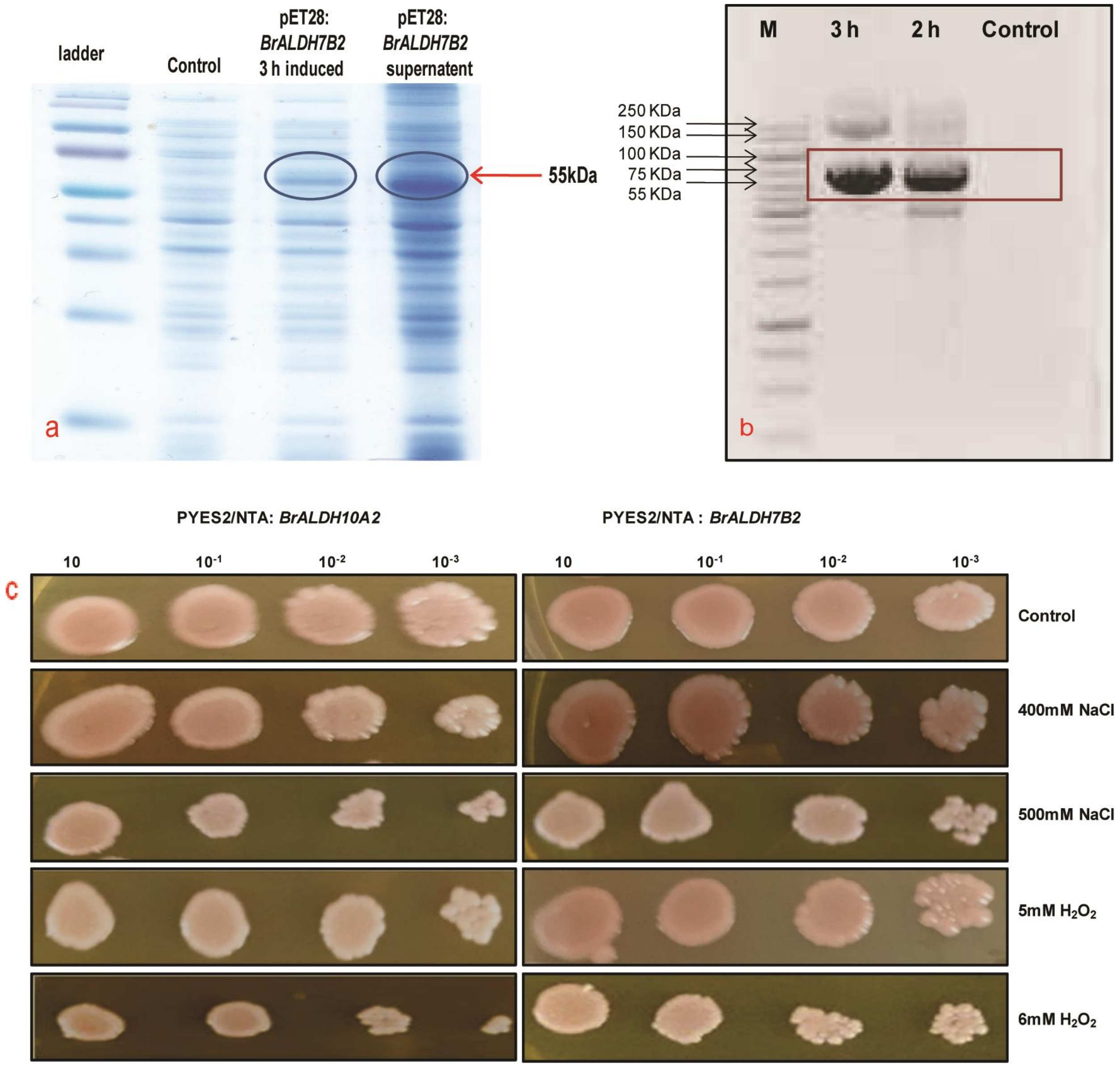

.
